# Ecodesign of Kesterite Nanoparticles for Thin Film Photovoltaics at Laboratory Scale

**DOI:** 10.1021/acssuschemeng.4c02841

**Published:** 2024-07-26

**Authors:** Michael D. K. Jones, Bethany L. Willis, Stephen Campbell, Giray Kartopu, Pietro Maiello, Prabeesh Punathil, Wai Ming Cheung, Elliot Woolley, Lewis C. R. Jones, Ochai Oklobia, Adam Holland, Vincent Barrioz, Guillaume Zoppi, Neil S. Beattie, Yongtao Qu

**Affiliations:** † Department of Mathematics, Physics and Electrical Engineering, Northumbria University, Newcastle upon Tyne, NE1 8ST, U.K.; ‡ Department of Mechanical and Construction Engineering, Northumbria University, Newcastle upon Tyne, NE1 8ST, U.K.; § Wolfson School of Mechanical, Electrical and Manufacturing Engineering, Loughborough University, Loughborough, Leicestershire LE11 3TU, U.K.; ∥ Centre for Solar Energy Research (CSER), in the Centre for Integrative Semiconductor Materials (CISM), Faculty of Science and Engineering, Swansea University, Bay Campus, Swansea, SA1 8EN, U.K.; ⊥ HORIBA UK Limited, Kyoto Close, Moulton Park, Northampton, NN3 6FL, U.K.

**Keywords:** ecodesign, kesterite, CZTS, LCA, photovoltaics, nanoparticles, sustainability

## Abstract

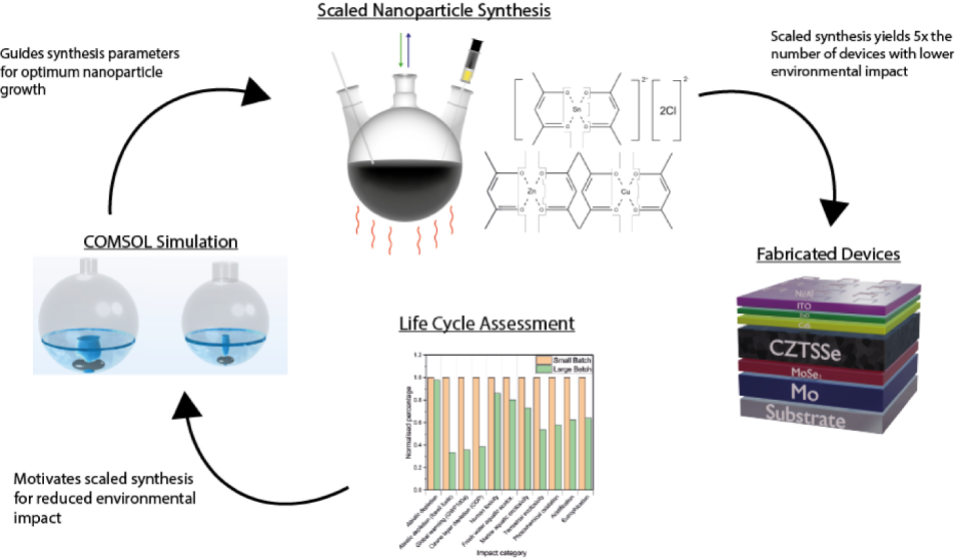

This manuscript investigates the efficient synthesis of copper zinc tin sulfide (CZTS) nanoparticles for CZTS thin film solar cell applications with a primary focus on environmental sustainability. Underpinning the investigation is an initial life-cycle assessment (LCA) analysis. This LCA analysis is conducted to evaluate the environmental impact of different synthesis volumes, providing crucial insights into the sustainability of the synthesis process by considering the flows of material and energy associated with the process. Life-cycle assessment results demonstrate that significant reductions to the environmental impact can be made by increasing the synthesis volume of CZTS nanoparticle ink. Using a 5-fold increase in volume can reduce all 11 investigated environmental impacts by up to 35.6%, six of these impacts demonstrating reductions >10% and the amount of global warming potential is reduced by 21.4%. Motivated by the LCA results, COMSOL simulations are employed to understand the fluid flow patterns in large-scale fabrication. Various sizes and speeds of stirrer bars are investigated in these simulations, and it is determined that a 50 mm stir bar at 200 rpm represents the optimal configuration for the synthesis process in a 500 mL flask. Subsequently, large-batch CZTS nanoparticle inks are synthesized using these parameters and compared to small-batch samples. The light absorbers are characterized using Raman spectroscopy and X-ray diffraction, confirming favorable properties with close-to-ideal elemental ratios in large-batch synthesis. Finally, solar cell devices fabricated utilizing CZTSSe absorbers from the large volume synthesis process demonstrate comparable performance to those fabricated using small-batch synthesis, with uniform power conversion efficiencies of around 5% across the substrate. This study highlights the potential of large-volume CZTS nanoparticle synthesis for efficient and environmentally friendly CZTS solar cell fabrication, contributing to the advancement of sustainable renewable energy technologies.

## Abstract 2

The sustainable scaling of the kesterite nanoparticle synthesis process with environmental impact and chemical waste is described through a life-cycle assessment.

## Introduction

With significant cost reduction over the past decade, large-scale solar farms and roof-mounted domestic photovoltaics (PV) are now much more widespread. Cumulative global PV installation reached 1 TW in 2022,^1^ with an annual growth rate of cumulative PV installations of 30% between 2011 and 2021. Continuation of this growth is crucial in order to meet the aims of the Paris Agreement and limit global warming to within 1.5 ^◦^C.^2^ However, the contribution from these traditional silicon-based solar panels to electricity production is still limited to only 3.6% of the world’s electricity share.^3^ To meet the ambitious global target to increase PV capacity by 75% by 2027,^4^ global production of 1 TW installations per year is expected by the end of the decade.^5^ Therefore, it is important to develop new PV technologies which can contribute to this goal and may also be more suited to application-specific implementation, e.g., building integrated photovoltaics.

Derived from copper-based chalcogenides, the quaternary compound kesterite Cu_2_ZnSnS_4_ (CZTS) is composed of naturally abundant (crust abundance of 68, 79, and 2.2 ppm for copper, zinc, and tin, respectively) and relatively cheap elements,^6,7^ making it an attractive alternative PV material. CZTS has a near optimum direct energy bandgap of 1.5 eV and a large absorption coefficient of 10^4^ cm^–1^ (in the photon energy range greater than 1.2 eV),^8,9^ minimizing the thickness of material needed to absorb the incident light and hence further reducing material costs. More importantly, kesterite is compatible with novel solution processing technologies, further underpinning it as an appealing candidate for large-scale TW PV module manufacturing with relatively low energy consumption and cost.^10,11^

Among the variety of solution-based techniques, nanocrystal coatings provide the only approach that enables the phase formation to occur prior to the film deposition, which is critical for this quaternary compound to achieve a thermodynamically stable phase for high-performance solar cells.^12,13^ Furthermore, the post-synthesis washing treatment in the nanocrystal-based method can remove most of the residual chemicals and provide air-stable inks for thin film depositions.^14−16^ The deposition of CZTS from nanoparticle inks and subsequent annealing therefore offers a low-cost route to fabricate high-quality absorber layers and solar cells.^17−19^ However, nanoparticle inks for solar absorber applications have traditionally been fabricated at a small scale and only limited inks of a few milliliters could be used for the following thin film deposition.^20−22^

Despite the relatively small volume of material used in research-scale CZTS photovoltaics, the absorber layer has been identified as a hotspot of environmental impact^23^ and we therefore perform a “cradle-to-gate” life-cycle assessment (LCA) to understand the relative change in environmental impacts when the synthesized volume of nanoparticle ink is increased by a factor of 5. A glossary has been added to the Supporting Information to aid in understanding the LCA terminology and impact categories included in this study. LCA can be used to compare similar PV devices,^24^ different PV technologies,^25^ or manufacturing processes of common cell structures.^26^ In general, across all PV technologies, efficiency, lifetime, and energy consumption during manufacture are key variables which strongly affect the overall environmental impact. Despite containing more “harmful” and “scarce” materials, CuInGa(S,Se)_2_ (CIGS) has a lower environmental impact than other chalcogenide thin film solar cells because less material is needed compared with less efficient technologies to generate 1 kWh of electricity.^24^ However, when the cells were compared assuming identical efficiencies, CIGS was found to be the most harmful for the environment since it had the greatest value in all impact categories (except abiotic depletion), while Sb_2_Se_3_ cells were found to have the lowest impact.^27^ Similarly, research into the manufacturing process of perovskite solar cells found that the environmental impact was less than that of silicon,^26^ but the impact over the whole life cycle for perovskites was greater than the impact for all other commercially available PV technologies due to the shorter lifetime of modules.^26,28^

Compared to crystalline silicon PV, thin-film technologies are generally more environmentally friendly and CZTS was found to have the lowest environmental impact among other studied emerging thin-film technologies.^29,30^ However, a comparison between CZTS and antimony-based thin films found antimony-based cells to have lower impacts than CZTS and research investigating Zn_3_P_2_ (an alternative to CdTe) and CZTS (as an alternative to CIGS) found that CdTe and Zn_3_P_2_ each had lower impacts than CZTS and CIGS.^23^ CZTS has also been investigated as a top cell for tandem cells where the CZTS/perovskite tandem cells have been identified as among the lowest environmental impact of all investigated combinations; however, it is limited by its low power conversion efficiency making perovskite/perovskite tandems more favorable.^31^ Often, when CZTS PV is investigated, the electricity is found to be the hotspot and contributes more to the overall impact than the materials do. One report indicates that future PV technology development should focus more on process improvement in order to reduce the environmental impact. It has also been identified that for CZTS technology the absorber layer is the most environmentally demanding stage.^23^

There are several methods which may be used to quantify environmental impact and a review of these can be found in the literature.^32^ Some of these methods only investigate a small range of environmental impact categories, whereas LCA considers a much larger range, providing a better representation of the whole environment. Some of them also consider only the contribution of materials, emissions, and/or energy separately, whereas LCA considers all three flows simultaneously to determine the impact on the environment. Some of them, such as exergy analysis, do not directly quantify the environmental impact but point only to insights where improvements could be made. LCA also has defined international standards (ISO 14040/ISO 14044) which several of the other methods do not. On this basis, LCA was selected as the methodology for this study.

The approach we present here uses LCA at the beginning of the research process to guide the experimental design for scaling up nanoparticle ink synthesis. Although the increased volume of CZTS nanoparticle ink remains at the laboratory scale, this results in a more sustainable process. Based on this advantage, we then analyze the effect of upscaling on the reaction conditions, the resulting material properties, and the device performance. Simulations of the ink synthesis process were performed in 2D and 3D, which informed us about suitable conditions for the scaled-up synthesis. We observe no degradation in the PV performance relative to the smaller ink volume and highlight the opportunity to use initial LCA and COMSOL simulations to guide the scaling of the synthesis process efficiently.

## Results and Discussion

### Life-Cycle Assessment

We performed a “cradle-to-gate” LCA (life-cycle assessment) to investigate the environmental impact of scaling up the chemical synthesis of CZTS nanoparticle ink from 20 mL (“small batch”) to 100 mL (“large batch”) in 100 and 500 mL volume flasks, respectively. The LCA has been used to quantitatively assess the impact of different nanoparticle and nanoparticle ink volumes to make comparative judgments about their sustainability. The goal of the LCA is to understand the relative change in environmental impact categories caused by scaling-up of the volume of ink synthesis. Although the LCA is restricted to the laboratory scale, this is an important step in considering the sustainability of ink synthesis for larger-scale PV cell fabrication in addition to exploring the potential for reducing the environmental impact of PV device research. A limitation of the LCA is that it is restricted to the CZTS nanoparticle ink synthesis, which is only one stage of the PV device fabrication. The justification for this is that we ultimately perform a relative comparison of the device performance (small vs large batch) in which all other fabrication steps were kept identical.

**Figure 1:**
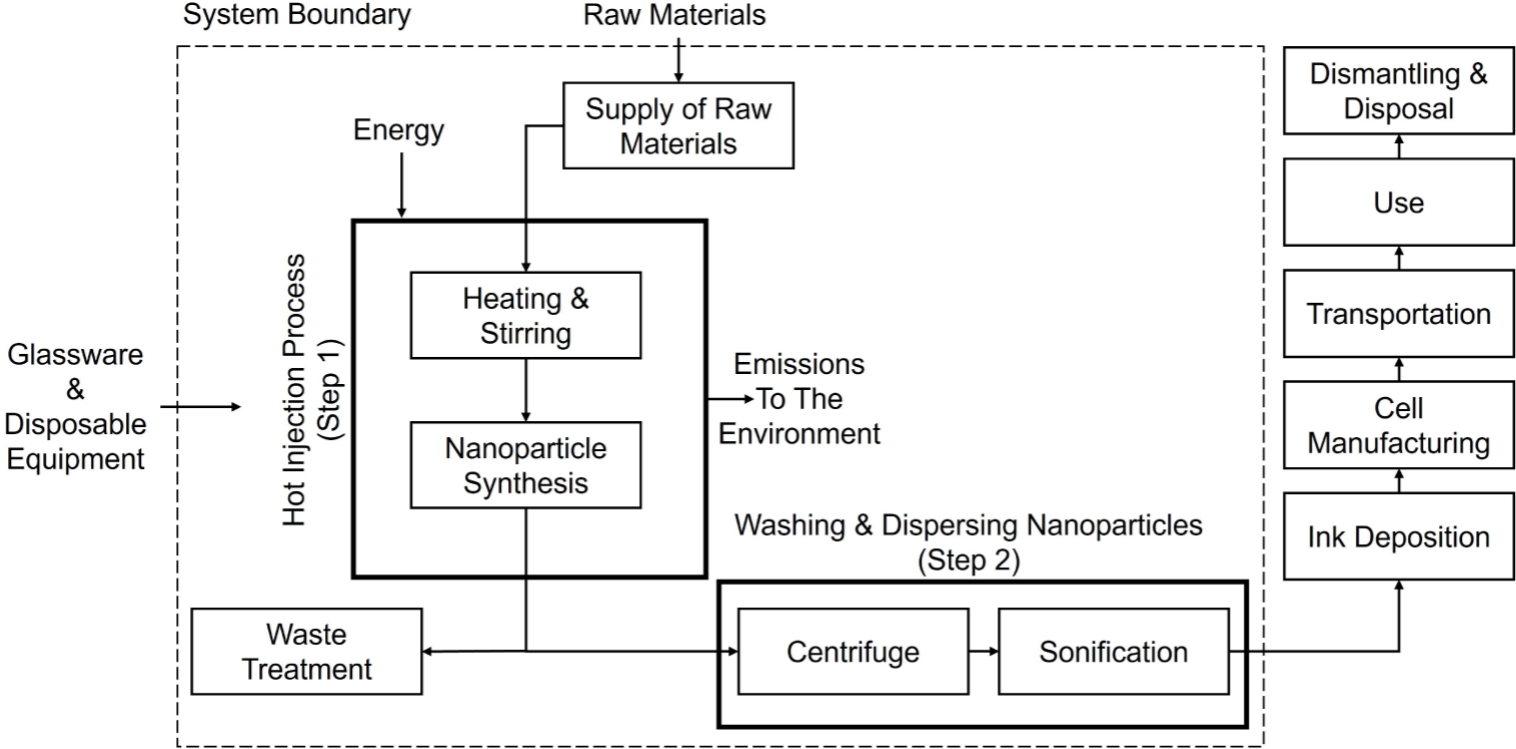
System boundary illustrating the limits of the LCA and highlighting the unit process investigated for this study.

The scope of the LCA is the chemical synthesis of CZTS nanoparticles and their washing and dispersion into a nanoparticle ink used to create the thin film photovoltaic light absorber for use in research-grade solar cells. The investigation uses global market assumptions based on primary laboratory data collected at Northumbria University in the United Kingdom (UK) at laboratory scale in 2023. The functional unit is 20 mL of CZTS nanoparticles which are >5 nm diameter and of appropriate composition (Cu-poor, Zn-rich) produced during a single chemical reaction and prepared into nanoparticle ink for the fabrication of thin film PV light-absorbing layers. The system boundary is shown in Figure 1 and incorporated colloidal synthesis of CZTS nanoparticles via hot injection of sulfur into hot metallic precursors.^22^ All LCA was carried out according to “ISO 14040” and “ISO 14044” and to ensure that this LCA is replicable, the LCI has been included in the Supporting Information document.

LCA inputs were determined directly by measuring the masses of the nanoparticle ink reaction precursors given in the experimental section. In the absence of “oleylamine” (OLA) in the Ecoinvent v3.8 database, “tall oil, crude” was used as an approximation due to the relative similarity of the synthesis. The fatty acid fraction of tall crude oil contains a large amount of the OLA obtained through fractional distillation. Electricity consumption was measured directly using a Multicomp Pro power meter during nanoparticle synthesis.

Creation of the CZTS nanoparticle ink can be considered as a 2-part process—the first step being the synthesis of the CZTS nanoparticles through a hot injection process as identified in Figure 1 within the system boundary, the second step being the cleaning and dispersion of these nanoparticles using solvents to prepare the nanoparticle ink also identified in the system boundary.

**Figure 2:**
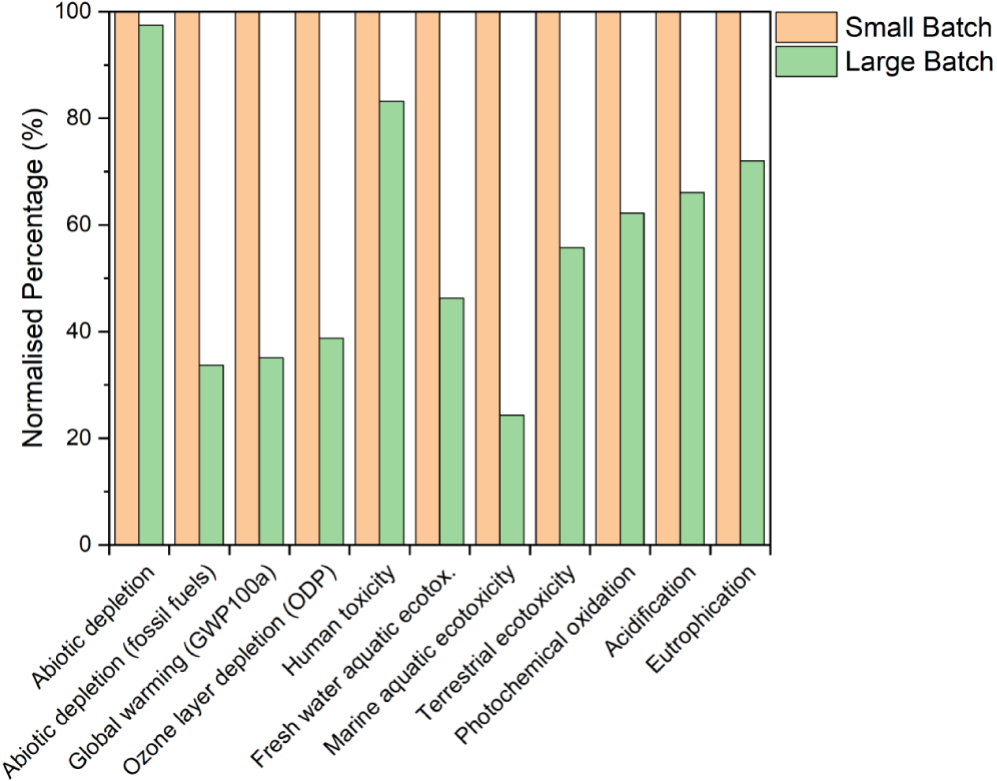
Normalized impact of different environmental categories for small-batch and large-batch nanoparticle synthesis.

Based on the raw data in Table S2, Figure 2 shows the LCA results normalized to the maximum value for each impact category for the nanoparticle synthesis only (i.e., the first step). Relative to the small batch, the large-batch synthesis exhibits lower impacts across all investigated impact categories and more than a 25% reduction across 9 of the 11 impact categories. The difference in the environmental impact of the materials alone was found to be less than the impact of the electricity consumed during the nanoparticle synthesis process for some impact categories which is consistent with other works.^24^ Considering all inputs, large-batch fabrication of the nanoparticles can cause a decrease between 2.6% (in abiotic depletion) and 75.7% (in marine aquatic ecotoxicity) compared to small-batch fabrication in the investigated environmental impact categories. The difference between large batch and small batch for abiotic depletion, human toxicity, and eutrophication was found to be the smallest because the materials dominating these impacts were ones that were used in the same ratios when scaled up so it is expected that these impacts are similar. The greater differences between batch sizes for each of the other environmental impact categories are most likely due to the lower amount of electricity consumed and reduced amount of waste for the large batch (per 20 mL) in comparison to the small batch. Further analysis of the results indicated that the most frequent highest contribution from each input across the impact categories was found to be (in decreasing order): electricity consumption, copper, OLA, tin, waste treatment, zinc, and sulfur. The amount of waste being treated dominated marine aquatic ecotoxicity and freshwater ecotoxicity, with marine aquatic ecotoxicity showing the greatest difference in impact between batch sizes. This difference in impact may change significantly depending on the type of waste treatment used in the LCA, and here it has been chosen to be municipal treatment by incineration in the UK.

### LCA of Nanoparticle Ink Fabrication

**Figure 3:**
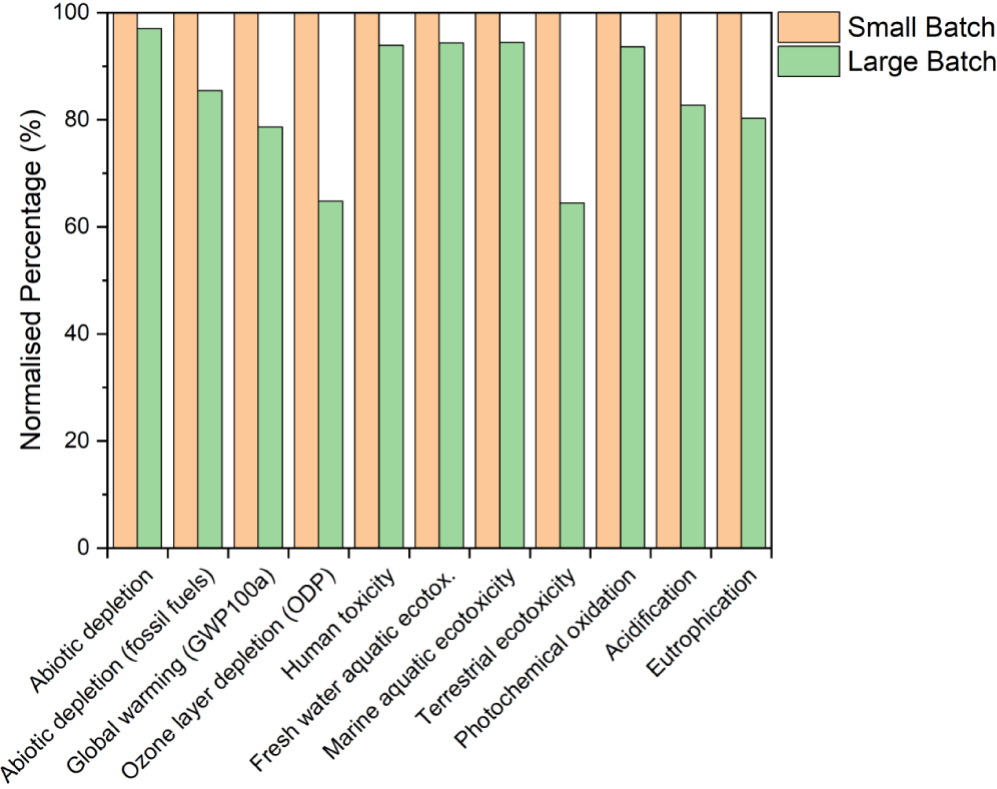
Normalized impact of different environmental impact categories for small-batch and large-batch nanoparticle ink synthesis.

Following the chemical synthesis of the nanoparticles, it is necessary to wash them using solvents such that they are suitable for deposition to form a thin film PV absorber. The use of these solvents is an important part of producing nanoparticle ink and has associated environmental impacts. The LCA has therefore been extended to include this step and Figure 3 shows the results. Considering the production of the nanoparticle ink (rather than just the nanoparticle synthesis), the difference in impact between small batch and large batch is reduced but still shows that synthesizing ink in large-batch volume is environmentally better than using a small batch. This smaller environmental difference between ink synthesis volumes is the result of the linear scaling of the mass of solvents used for each batch size, making the environmental benefits from the difference in nanoparticle synthesis volumes and the difference in electricity consumption during cleaning and dispersion a smaller proportion of the overall impact for each impact category.

All 11 environmental impact categories in Figure 3 show a decrease when using a large batch compared to a small batch, with 6 of these showing greater than 10% decrease in impact. The average percentage decrease between small batch and large batch across the 11 environmental impact categories was found to be 15.5% with the greatest difference being 35.6% for terrestrial ecotoxicity which is dominated by OLA, electricity consumption, and copper—all of which are also involved in the nanoparticle synthesis process (step 1). Additionally, the amount of electricity consumed for the large batch during cleaning and dispersing of the nanoparticles (step 2) is just 40% per 20 mL of the amount of electricity consumed in the small batch resulting in a greater difference between the batches for this impact category. The smallest improvement from scaling up was only 3% for abiotic depletion which is dominated by tin and copper. Both of these metals are used in the creation of the nanoparticles and are used in equal ratios when scaling up so are equal per 20 mL and are not expected to differ in contribution to the impact of small-batch and large-batch synthesis when scaling up. Further analysis of the results indicated that the most frequent highest contribution from each input across the impact categories was found to be (in decreasing order): electricity consumption, IPA, copper, waste treatment, OLA, tin, toluene, zinc, and sulfur. IPA was seen to be the most harmful to the environment in 5 of the investigated impact categories highlighting it as a priority in reducing the overall impact. This finding validates the use of alternatives to IPA in the production of CZTS nanoparticle inks using “green solvents” such as ethanol^33^ and motivates further work in the dispersion of other nanoparticle material systems. Analysis of the absolute data (shown in Figure S3) from the LCA reveals that marine aquatic ecotoxicity and freshwater aquatic ecotoxicity are responsible for the majority of the overall impact of the nanoparticle ink fabrication process. While this needs to be contextualized relative to a full device LCA, it implies that appropriate waste management strategies will be required to reduce any negative impact on living organisms in either freshwater or marine environments as a result of this type of solar cell fabrication. Relative to these impact categories, Figure S3 indicates that the absolute impact of the other environmental impact categories is not significant.

Assuming this nanoparticle ink is made into a product produced in a large batch at Northumbria University, UK, the carbon footprint of the 100 mL product is 1.45 kg CO_2_ eq (290 g CO_2_ eq per 20 mL) which can be used to coat 675 devices leading to 2.15 g CO_2_ eq. If a small batch had been used, a 20 mL product is 369 g CO_2_ eq which can coat 135 devices leading to 1.8 g CO_2_ eq per device at the laboratory scale. This is 0.58 g more per device from using small-batch ink than when using a large batch, which is more than a 21% increase in CO_2_ eq. These devices are 4 mm × 4 mm, 0.16 mm^2^ area.

### Impact of Electricity Mix on Nanoparticle Ink Fabrication

**Figure 4:**
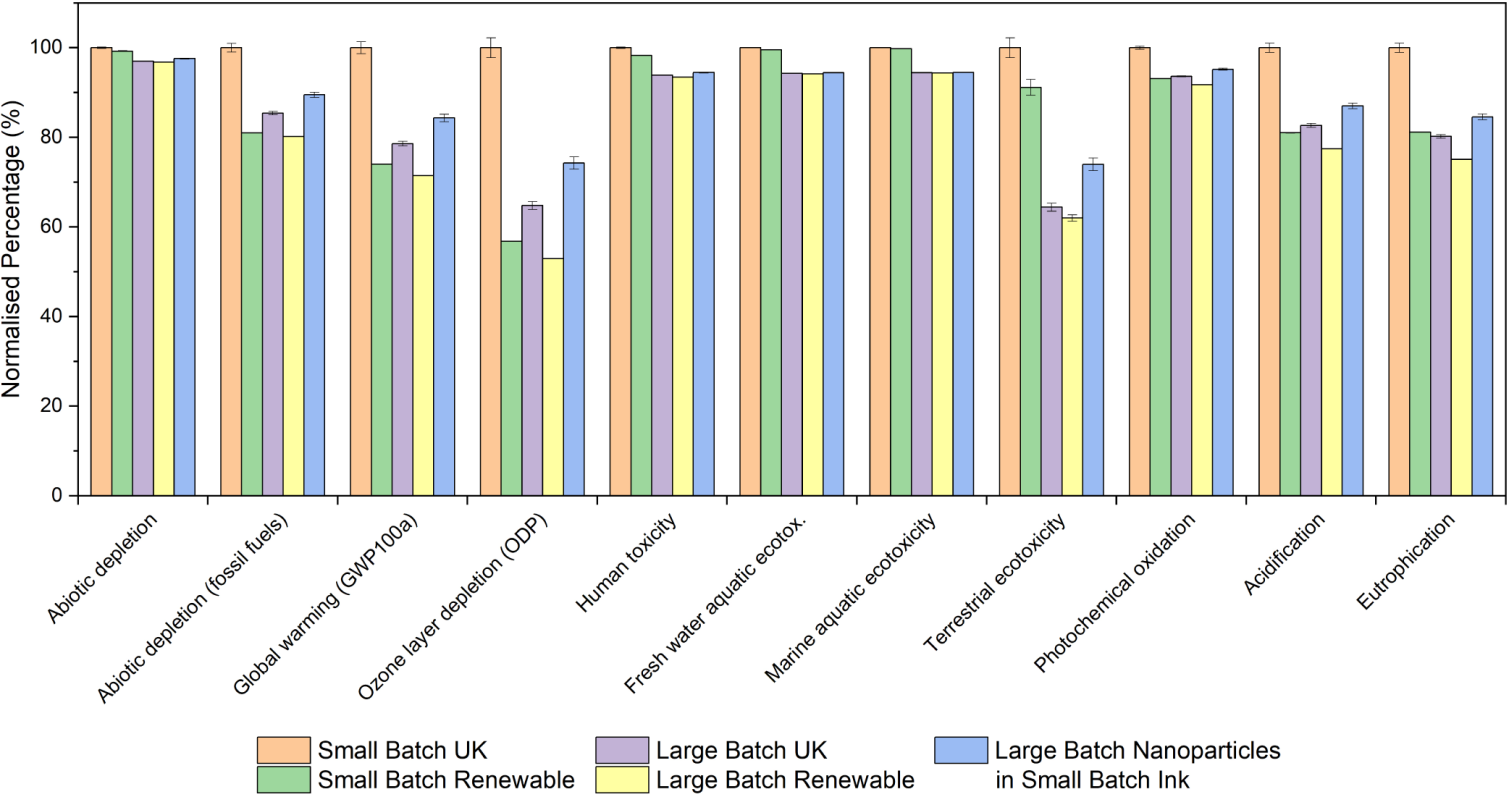
Normalized impact of different environmental impact categories for nanoparticle ink synthesis modeled using a UK electricity mix for small-batch and large-batch, renewable resource-based electricity mix for small and large batches, and UK for large-batch nanoparticles incorporated into small-batch ink synthesis.

Since electricity was found to play a significant role in reducing the environmental impact when scaling up to a large batch, an entirely renewable electricity mix has been applied and investigated to the fabrication of a small batch in the LCA. Taken from the Ecoinvent v3.8 database, the sole use of renewable energy resources has been assumed by adopting the “renewable energy products” electricity mix model taken from Switzerland. The results from this are shown in Figure 4 and the raw data can be found in Table S4. These show large-batch nanoparticle ink can be better environmentally across 6 of the investigated environmental impact categories. As expected, the impact of using a UK-based electricity mix to fabricate a small batch of nanoparticle ink has the greatest impact on all of the investigated environmental impact categories. A more in-depth analysis of the contribution of different types of renewable resources to the electricity mix and the origin of each of their impacts would be beneficial in understanding the true cause of impact from this modeled electricity mix, which lies beyond the scope of investigation in this work. The reduction in impact from using a renewable energy-based electricity mix for small-batch fabrication in comparison to the UK electricity mix results in an average decrease of 13.1%.

Fabricating a large batch of nanoparticle ink using a UK electricity mix was found to reduce the environmental impact in 6 impact categories even more with a further reduction, on average, of 7.5% across the 11 categories compared to fabricating a small batch using renewable energy. To further explore the impact of renewable electricity and the impact of scaling up, a small batch of ink is fabricated using nanoparticles synthesised from a large batch (scaling up part 1 of the process only) and the results are compared to fabricating a small batch using a renewable electricity mix. The results show that scaling up step 1 (the nanoparticle synthesis/ hot injection process) only can result in a decrease in all but 5 environmental impact categories compared to fabricating a small batch using renewable electricity demonstrating that scaling up part of the process can have environmental benefits comparable to using a renewable electricity mix.

### Cost Implications of Upscaling

In addition to environmental impact, we analyzed the economic impact of scaling up the nanoparticle ink synthesis volume. Table S1 compares the cost of both processes considering the raw materials, laboratory equipment, electricity usage, labor, and waste disposal costs. For the raw materials, large-batch synthesis is proportionally more expensive. However, the difference in the cost of equipment and electricity is much smaller while the labor and waste disposal costs are approximately the same. Taken together the large-batch ink costs £357.98 per 20 mL versus £1477.28 per 20 mL for the small batch. Although these figures are based on laboratory quantities (rather than industrial), they point toward a ∼75% economic saving using large-batch fabrication.

### Simulation of the Synthesis Process

As studied in the LCA, large-batch CZTS nanoparticle ink fabricated in a 500 mL flask is preferred for an overall lower environmental impact. In the nanoparticle colloidal synthesis, a Teflon-coated magnetic stir bar is used to agitate the solution and provide efficient mixing for uniform CZTS nucleation and nanoparticle growth. Nucleation is believed to be the first step in the classical crystallization model.^34^ It describes the process where tens to hundreds of atoms/monomers (“nanoclusters”) are formed as the seed for establishing the following molecule-to-solid transition.^35^ Maintaining a level of supersaturation is crucial in generating short bursts of nucleation and producing uniformly sized nanoparticles.^36^ The stirring rate has to be controlled to achieve efficient nanoparticle growth; for example, too high a rate will increase fluid shear and restrict nucleation.^37^ Therefore, it is important to understand the influence of stirring on fluid flow patterns during nanoparticle synthesis. In this regard, COMSOL simulations were performed to describe the flow field generated by the magnetic stirrer^38−40^ inside the CZTS nanoparticle crystallization flask and provide parameter guidance for laboratory scale-up of CZTS nanoparticle inks.

The fluid flow field in a spherical 500 mL flask (150 mm diameter and 20 mm neck height) is simulated in this study. The volume of solvent used in the large-batch ink fabrication is replicated in the fluid dynamics simulation. Turbulent fluid flow dynamics in the flask were studied to understand the effects of increasing the size and stirring speed of the stir bar. Three sizes of stirrer bars with spherical end cups are considered at speeds of 100 and 200 rpm, respectively. The relevant geometrical parameters, including the length (*l*) and width (*w*) of the bars used, are summarized in Table 1.

**Table 1:** Geometrical Parameters of the Stirrer Bars

stirrer bar	length (mm)	diameter (mm)
A	33	15
B	50	20
C	65	20

**Figure 5:**
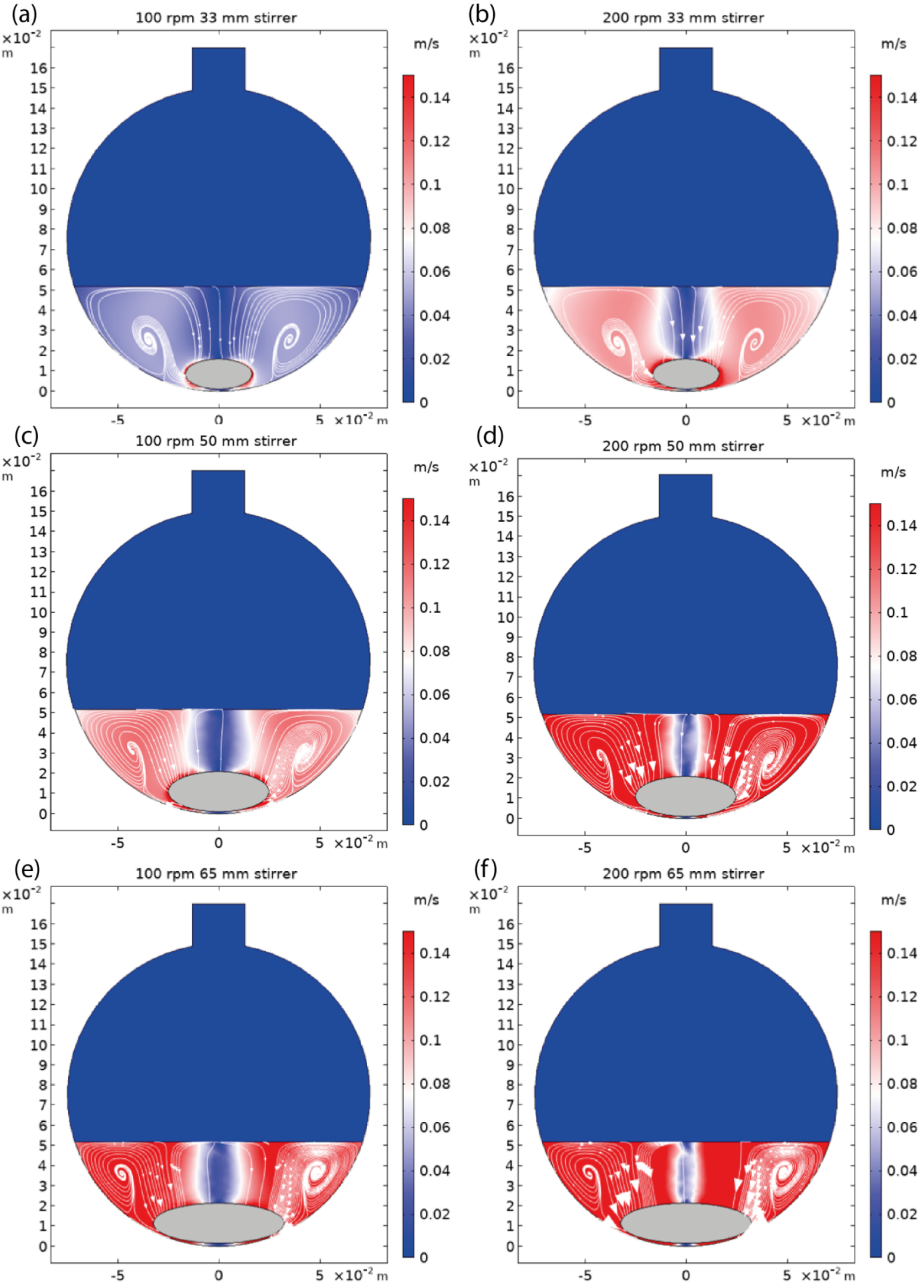
2D velocity magnitude patterns of the simulated flow field in a 500 mL flask using a stir bar length/stirring speed of (a) 33 mm/100 rpm, (b) 33 mm/200 rpm, (c) 50 mm/100 rpm, (d) 50 mm/200 rpm, (e) 65 mm/100 rpm, and (f) 65 mm/200 rpm. Color gradients from dark blue to red represent flow velocity from low (0 m/s) to high (0.15 m/s). The thin continuous lines represent streamlines of the velocity field.

The fluid flow patterns in the flask, represented as velocity magnitude and velocity field streamlines under different stirring conditions, are given in Figure 5. For all of these stirring conditions, two flow cells are formed in a given vertical slice of the flask, with the centers of two-dimensional vortices located roughly below half of the fluid height. It is clearly visible that the fluid circulation pattern is upwelling from the bottom and walls to the top of the fluid surface and then downwelling toward the stirrer in a narrow region on the symmetry axis of the flask.

**Figure 6:**
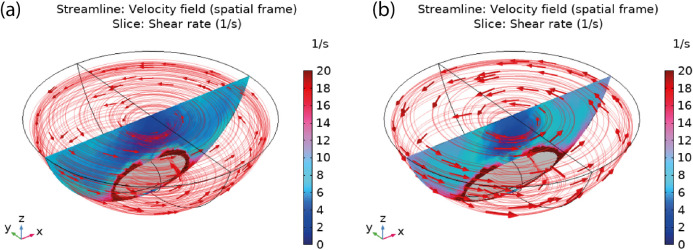
3-D simulation of the flow field in a 500 mL flask with (a) 50 mm and (b) 65 mm long stir bar at 200 rpm. The 2-D slices show fluid shear rate in the *xz* plane. The uniform velocity streamlines indicated by the red arrows in (a) correspond to a lower shear rate compared to (b) where the shear rate is higher due to uneven velocity distribution.

As shown in Figure 5a, a 33 mm long stir bar at 100 rpm can only yield a low-velocity flow field (<0.06 m/s) in the two flow cells, while the central downstream region has near-zero velocity. Upon increasing the stirring speed to 200 rpm, as expected, the velocity field is improved in the fluid, with velocity in the flow cell region almost doubling to around 0.1 m/s. However, a wide near-zero velocity region can still be observed in the middle of the flask, as shown in 5b. In Figure 5c,d, a higher and more uniform velocity field (≈0.14 m/s) can be clearly observed when using a bar of 50 mm length and 20 mm diameter. Importantly, the width of the low-velocity region in the middle of the flask significantly reduces when a 200 rpm stirring speed is used, indicating that the fluid is mixed properly. If an even bigger stir bar (65 mm in length, 20 mm in diameter) is applied, a higher velocity field can be achieved at a low stirring speed of 100 rpm and the low-velocity region becomes negligible when the stirring speed increases to 200 rpm as shown in Figure 5e,f. However, it is important to notice that as the size of the stir bar increases, the shape of the main vortices changes and becomes more elliptical. For the smallest stir bar, the vortices are nearly circular. Figure S3 shows the sensitivity of the shear rate for the 500 mL flask synthesis with rpm increases of 10 rpm from 100 to 200 rpm. This high stirring rate could generate fluid shear that is detrimental to CZTS nucleation by attrition of the nanoclusters.^37^ This is evident in Figure 6 where the shear rate is increased when a larger stir bar is used to mix the fluid at 200 rpm. In the simulation with a 50 mm stir bar, the velocity streamlines appear evenly distributed with uniform velocity magnitude compared with the 65 mm stir bar, which reduces the shear rate in the fluid. With all factors considered, a 50 mm stir bar at 200 rpm stirring speed would be the optimum for the CZTS nanoparticle fabrication in a 500 mL flask.

### CZTS Nanoparticle Ink Scale-Up Synthesis

Utilizing the recommended stirring parameters derived from the COMSOL simulation, a 50 mm stirrer at 200 rpm, we advanced to the large-scale fabrication of CZTS nanoparticle ink within a 500 mL flask. In order to draw a comparison, a small-scale injection synthesis was concurrently conducted in a 100 mL flask, as described in previous work.^22^ To maintain consistent molar concentrations between the two setups, five times the number of chemical precursors was introduced into the larger flask (refer to the experimental section for a comprehensive methodology description). Following a reaction period of 30 min at 225^◦^C, the resultant nanoparticles were subjected to precipitation and underwent two subsequent washes with isopropanol (IPA) and toluene. Subsequently, the collected CZTS nanoparticles were dispersed through sonication, yielding CZTS nanoparticle inks with a concentration of 100 ± 50 mg/mL, as detailed in our prior publications.^14,15,22^

**Figure 7:**
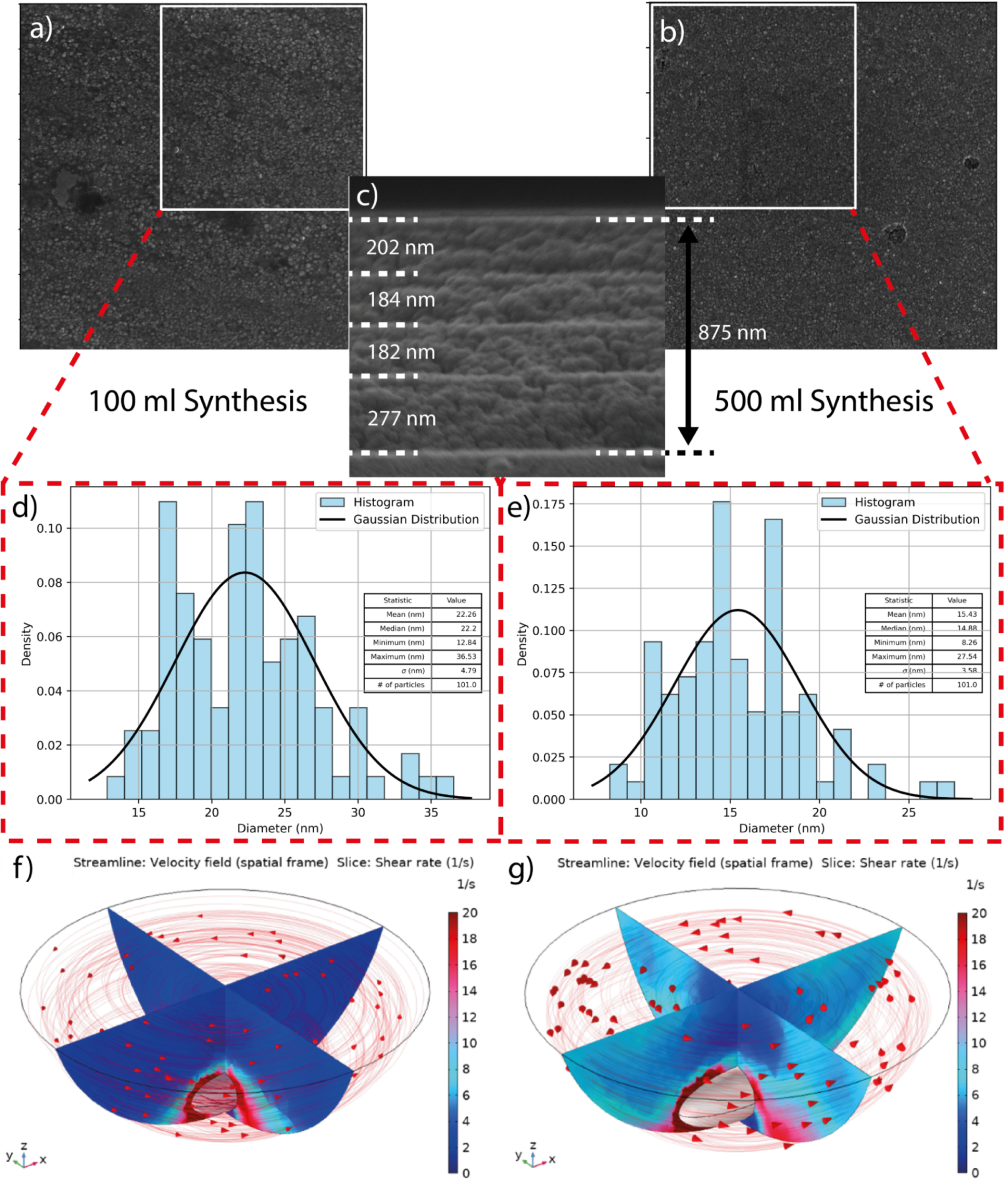
(a) and (b) show top-down SEM images of CZTS nanoparticle precursor films synthesized from the small and large batches, respectively. (c) A Cross-sectional SEM image of as-deposited CZTS layers from large-batch nanoparticles. (d) and (e) show histogram plots with Gaussian fits depicting nanoparticle diameters of small and large syntheses, respectively. 3-D simulation showing the velocity field in the fluid of (f) 100 mL flask with a 20 mm stirrer and (g) 500 mL flask with a 50 mm stirrer. For comparison, the arrowheads are proportionally plotted in a log scale in order to show the lower velocity stream in the 100 mL flask. The 2-D slices in the *xz* and *yz* planes show a fluid shear rate where the shear rate is significantly higher in the bulk of the fluid in the 500 mL flask compared to the 100 mL flask.

Following the nanoparticle synthesis, four layers of concentrated CZTS nanoparticle inks suspended in hexanethiol were deposited via a slot die coating. Figure 7a shows the top-view SEM image of the as-deposited CZTS nanoparticle thin film. It can be observed that the film is smooth and is formed of densely packed, spherical nanoparticles. Nanoparticle size and coverage are homogeneous and consistent over the 25 × 75 mm^2^ samples. Large-batch fabrication yields a smaller mean nanoparticle size at 15.43 nm, compared to 22.26 nm for the small-batch synthesis. Nanoparticle size was calculated using top-down SEM images of as-deposited films, with approximately 100 nanoparticles being selected to contribute to a histogram distribution of diameters with Gaussian fit, depicted for a small batch (Figure 7d) and a large batch (Figure 7e). As studied in the COMSOL simulation in Figure 7 f,g, the high stirring rate and fluid shear observed in 500 mL synthesis are believed to attrite clusters/small crystals and lead to smaller nanoparticles.^37^ These nanoparticle diameters are also in agreement with the crystallite size calculations from XRD pseudo-Voigt profile peak fitting conducted on the three main peaks of the CZTS kesterite pattern at 2θ angles 28.5°, 47.5°, and 56.25°. The average crystallite sizes for small and large batches are 14.31 and 17.76 nm, respectively (Table S5). It is important to note that the Scherrer equation returns domain/crystallite size which does not necessarily correlate to particle or grain size.^41^

Despite the morphological differences, the nanoparticle composition was comparable between the two synthesis processes. Table 2 shows the atomic percentages of Cu, Zn, Sn, and S as well as elemental ratios. Ideal elemental ratio ranges have previously been defined as Cu/(Zn + Sn) = 0.75–0.85 and Zn/Sn = 1.05–1.25.^22^ Our nanoparticle composition falls in the region of slightly Zinc poor and Sn rich.

**Table 2:** Composition and Atomic Ratios of As-Deposited CZTS Thin Film from Control and Large-Batch Synthesis, Respectively^a^

element	small volume synthesis (at %)	large volume synthesis (at %)
Cu	22.11	22.01
Zn	13.59	13.70
Sn	15.92	15.83
S	48.37	48.47
Cu/(Zn + Sn)	0.75	0.75
Zn/Sn	0.85	0.87

a Compositions were calculated by using EDX spectroscopy. The summation of Cu, Zn, Sn, and S atomic % (at%) was evaluated as 100%.

As shown in Figure 7b, the film is of uniform thickness, and a single slot die deposition can provide a thin film thickness of approximately 211 ± 39 nm. Four subsequent layers, as shown in Figure 7b, were deposited to achieve a total thickness of 900 nm for efficient light absorption. The CZTS precursor films undergo a selenization treatment described in more detail in the methodology section and past work.^14^ The selenization treatment converts the CZTS nanoparticle precursor films to CZTSSe micrometer-sized absorber layers to minimize the interface numbers.

**Figure 8:**
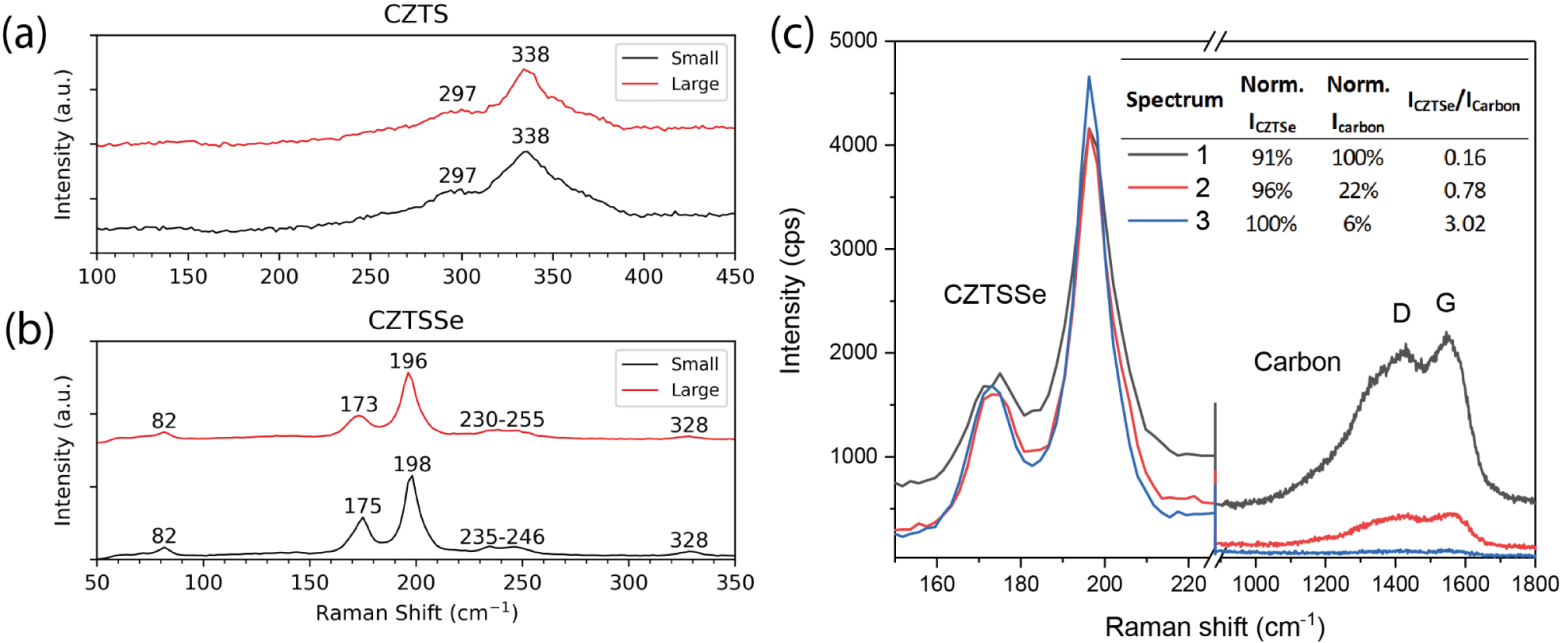
Raman spectra of (a) CZTS (as-deposited) and (b) CZTSSe (selenized) thin films made from small-batch and large-batch nanoparticle inks. (c) Raman spectra collected from a single CZTSSe thin film made by the large-batch nanoparticle ink. Inset compares the areal intensity of the main CZTSSe peaks and the carbon bands (D-like at 1400 cm^–1^ and G-like at 1550 cm^–1^).

To further investigate the structure and composition of the thin films, microRaman spectra were collected using multiwavelength excitations and analyzed. Figure 8 compares the first-order Raman modes observed using 532 nm excitation in as-deposited (CZTS) and selenized (CZTSSe) films made using small- and large-batch nanoparticle inks. The selenization process consists of a 20 min 500 ^◦^C anneal in a selenium atmosphere of partial pressure 130 mbar. The sample is then brought back to ambient temperature with fan-assisted cooling over the next 60 min. The full process has been outlined in previous works.^14^

For CZTS films (Figure 8a), both spectra appear identical, featuring one asymmetrical broad band extending from the region of 200 cm^–1^ up to 400 cm^–1^, centered at 335 cm^–1^, and a smaller shoulder peak at 297 cm^–1^. The former band closely resembles the spectrum reported^42^ for 5 nm CZTS nanocrystals dispersed in various liquids, suggesting that the as-deposited CZTS material retains its isolated nanoparticle nature without sintering, even after thermal treatment applied at 300 °C for solvent evaporation. The asymmetrical peak broadening is indicative of phonon confinement, i.e., nanosize effects and relaxation of selection rules.^43^ The peak at 297 cm^–1^, however, cannot be attributed to any known CZTS-related vibrations^44^ but rather to the Cu_3_SnS_4_ (CTS) ternary phase,^45^ which reasons well with the Sn rich, Zn poor composition obtained with EDX spectroscopy. A secondary impurity phase, attributable to Cu_2–*x*_S,^46^ was also detected using 488 and 405 nm excitations (see Figure S11). Selenization steps appear to remove both secondary phases, resulting in a pure CZTSSe phase.

For CZTSSe films (Figure 8b), the spectra are nearly identical with all Raman bands, assignable to the CZTSe phase, except for a small peak at 328 cm^–1^ which will be explained below. The main bands observed at 198 and 175 cm^–1^ correspond to A1 symmetry.^47^ The peaks in the region of 230–255 cm^–1^ may correspond to a mixture of B/E modes.^21^ In accordance with our data, first-principles calculations predict a band at 235 cm^–1^ due to Cu–Zn vibrations and 81 cm^–1^ due to Cu–Sn and Cu–Zn vibrations in the kesterite CZTSSe structure.^48^ Finally, the peak at 328 cm^–1^ can be attributed to the CZTSSe phase with an S/Se ratio of less than 30%,^49^ suggesting there are regions remaining with incomplete S-to-Se transition from the selenization treatment. Using the method described by Dimitrievska et al.,^50^ we estimate the S/(S + Se) ratio as 0.19 and 0.21 for the small- and large-batch-produced CZTSSe films, respectively. The slight downshift (by up to 2 cm^–1^) of the main CZTSSe bands at 175 and 198 cm^–1^ for the large-batch film may be explained by a reduction in the Zn content and an enrichment in Cu compared with the small-batch film.

In addition to the CZTSSe-related peaks, the presence of diamond (D)-like and graphite (G)-like carbon bands in the 1100–1700 cm^–1^ range^51^ were observed in the selenized films. Figure 8c displays a series of Raman spectra collected from a single CZTSSe film produced by using the large-batch ink. Interestingly, an inverse relationship is observed between the intensity of the CZTSSe-related bands and that of the graphitic carbon bands. This suggests that higher crystallinity in CZTSSe is achieved in regions where graphitic carbon removal is more pronounced.

To further characterize the polycrystalline phases of selenized films from both synthesis volumes, crystallographic diffractograms were obtained through X-ray diffraction 2θ scans. Both diffractograms exhibited comparable crystal phases, with the kesterite CZTSSe reference pattern (PDF 052 0868) appearing at the main peak locations with two theta angles of 27°, 46°, and 53.5° (Figure S5).

### Device Characterization

**Figure 9:**
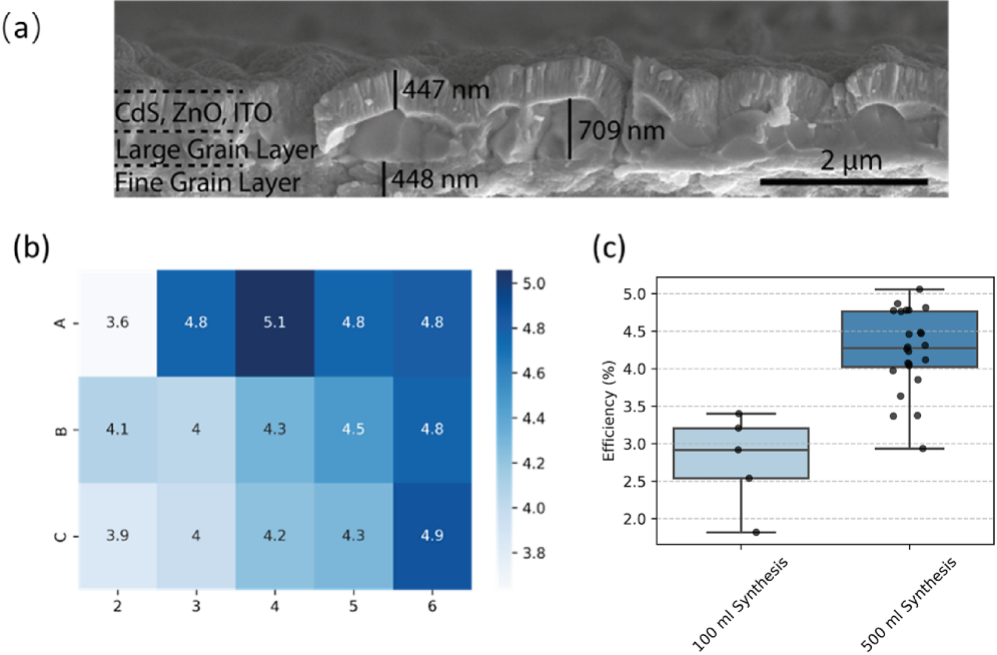
(a) Cross-sectional SEM image of a complete device showing the representative configuration including fine grain, large grain, and CdS, ZnO, and ITO layers. (b) Heat map distribution of power conversion efficiencies (unit: %) on a substrate fabricated from the 500 mL synthesis. (c) Box plot distribution of device performance comparison between small-batch and large-batch synthesis substrates.

The cross-sectional SEM image (Figure 9a) reveals a partial device structure showing only the top six layers, excluding the bottom Mo and Mo(S, Se)_2_ layers. The top layer, approximately 450 nm thick, is composed of the window layer for front conductivity and a CdS buffer layer to form the pn junction with the bottom CZTSSe absorber. The middle layer is about 700 nm thick and consists of densely packed micrometer-sized grains that contribute to light absorption and current generation. The bottom layer, approximately 450 nm thick, is a residual fine-grain layer believed to be carbon-rich and composed of byproducts from the large grain growth process. Reduced crystallinity in CZTSSe may be expected in this region, according to Figure 8c (1100–1700 cm^–1^ range).

The power conversion efficiency distribution of the completed devices is summarized in the heat map in Figure 9b, where each subrectangle represents a solar cell device with an area of 0.16 cm^2^. Apart from the upper left corner of the substrate, the efficiency is reasonably uniform across the substrate, with an average efficiency of around 4.3% using large-batch-fabricated CZTS nanoparticles. Figure S7 shows the *JV* characteristics of the champion cell synthesized at a large volume, with a power conversion efficiency of 5.06%, an open-circuit voltage of 414.31 mV, a short-circuit current density of 25.21 mA cm^–2^, and a fill factor of 0.48. Series and shunt resistance were also obtained as 4.55 Ω cm^2^ and 304.32 Ω cm^2^, respectively. The resistance observed in this study limited the device performance and further optimizations will improve the performance in component layers and interfaces in upscaling devices to match the device performance with ≥12% kesterite solar cells.^52^ From capacitance–voltage (*CV*) measurements, a built-in voltage of 456 mV and a depletion width of 59.32 nm were calculated, as depicted in the doping density vs depletion region width in Figure S8. EQE measurements in Figure S9 show a peak EQE of approximately 70% with a bandgap of 1.13 eV and an integrated *J*sc value of 23.78 mA cm^–2^, which is in line with the experimentally determined data from the *JV* measurement of 25.21 mA cm^–2^.

Inspection of the device performance distribution in Figure 9 indicates that a large-volume synthesis can provide comparable device performance with low environmental impacts compared to small-batch fabrication. A full JV parameter statistical comparison between synthesis volumes can be seen in Figure S10. Low-temperature photoluminescence (PL) measurements of small- and large-batch CZTSSe absorber samples were performed at varying excitation power densities to gain an insight into material properties of the absorber films to understand the significant increase in *V*_OC_ and the improved device performance in a large-batch sample.

**Figure 10:**
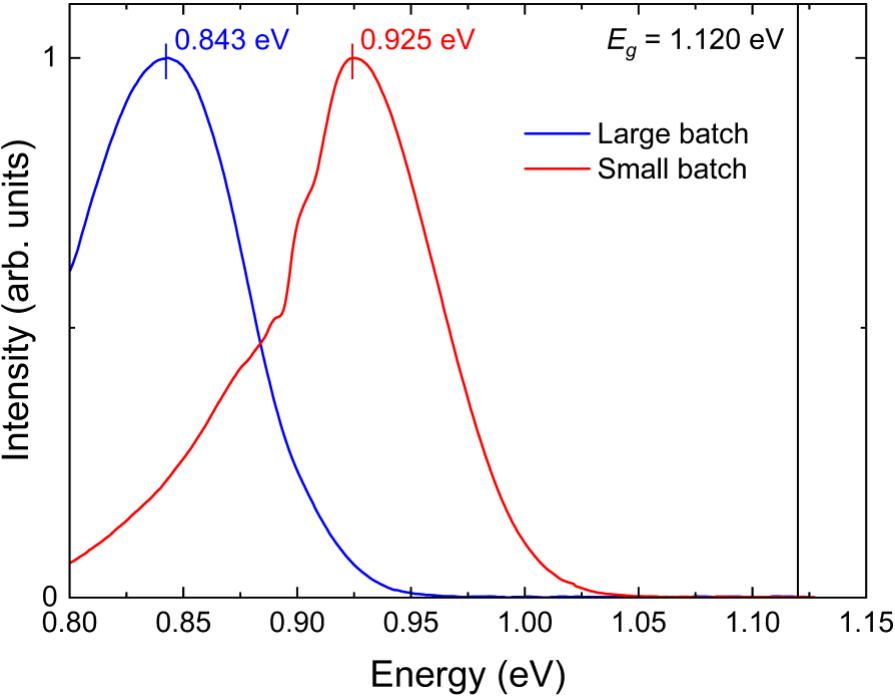
PL spectra of small- and large-batch CZTSSe absorber films at 6 K with the same 532 nm laser power density of 7.6 W/cm^2^. The solid line at 1.12 eV indicates the room temperature bandgap energy *E*_g_ of both absorbers. Note the fluctuation in the PL signal from the small-batch sample between 0.90 and 0.92 eV is due to water vapor absorption of EM radiation in that energy range.

CZTSSe is considered a highly doped and highly compensated semiconductor due to a high defect density such that the average distance *s* between defects is less than the defect Bohr radius.^53^ The presence of both donor and acceptor defects within the material bulk implies that the material is also highly compensated. The normalized 6 K PL spectra of small- and large-batch absorber samples at the same excitation power density (7.6 W/cm^2^) are shown in Figure 10.

The broad asymmetric shape of the PL bands for both types of absorbers indicates significant band-tailing in the materials. The PL peak maxima (*E*_PL_) are located at 0.925 and 0.843 eV for the small- and large-batch samples, respectively. The energy peaks are significantly redshifted from the corresponding bandgap energy *E*_g_ of 1.120 eV, and such a large redshift could be explained by the presence of deep defects within the bandgap. Tin-related acceptor defects such as V_Sn_, Cu_Sn_, and Zn_Sn_ have ionization energies in the range of 0.2–0.3 eV above the valence band maximum and could account for the observed redshift in both absorbers.^54^

**Figure 11:**
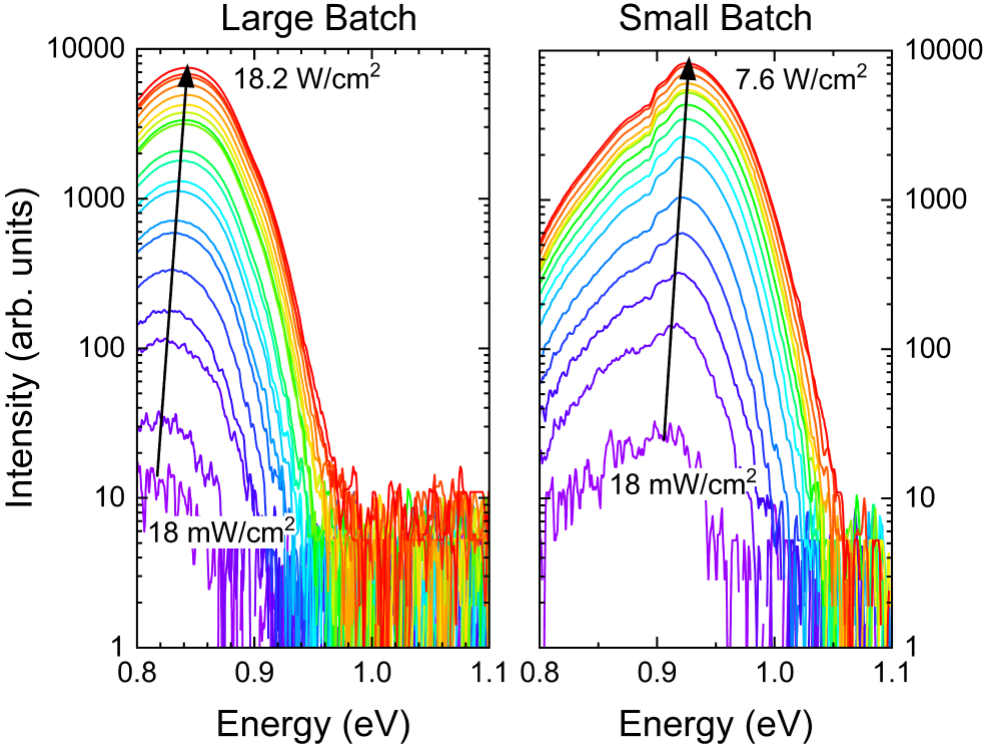
Excitation intensity dependence of the PL spectra with laser power *P* for large- and small-batch CZTSSe films at 6 K. Note the fluctuation in the PL signal from the small-batch sample between 0.90 to 0.92 eV is due to water vapor absorption of EM radiation in that energy range.

The low-temperature excitation-dependent intensity of the PL spectra for both absorbers is shown in Figure 11. In both samples, *E*_PL_ blueshifts in position with increasing power density until the PL signal saturates at laser power densities of 7.6 and 18.2 W/cm^2^ for the small- and large-batch films, respectively. Above the threshold intensity, *E*_PL_ does not increase and a high-energy shoulder appears upon increasing laser power (not shown in Figure 11 for clarity).

Gershon et al.^55^ observed similar behavior in CZTS films studied and attributed such phenomena to the presence of quasi donor–acceptor pair (QDAP) defects.^55^ The QDAP model describes the distribution of radiative donor–acceptor states that contribute to electrostatic potential fluctuations of the conduction and valence band energy levels in a semiconductor. The spectral position of the QDAP PL maximum *E*_PL_ can be described by
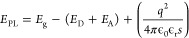
1where *q* is the elementary charge, ϵ_0_ is the vacuum permittivity, ϵ_r_ is the relative permittivity (or dielectric constant), *s* is the separation distance between donor and acceptor, *E*_D_ and *E*_A_ are the donor and acceptor energy levels separated from the conduction band minimum and valence band maximum, respectively. The last term in eq 3 describes the Coulomb potential that exists between donor and acceptor. The QDAP density *N*_D_ can be estimated from the maximum blueshift in *E*_PL_:^53^
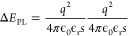
2
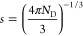
3An energy blueshift of magnitude of 33 and 29 meV was determined for small- and large-batch samples, respectively. Values of *N*_D_ for small- and large-batch films were calculated to be 1.87 × 10^17^ and 1.36 × 10^17^ cm^–3^, correlating to an average defect separation *s* of 5.21 and 5.80 nm (see Table 3). Band tailing in kesterites is manifested as a consequence of spatial inhomogeneities in the absorber material leading to fluctuations in the bandgap, clusters of charged defects creating fluctuations in the electrostatic potential or thermal vibrations of the crystal lattice (Urbach tails).^53^ Siebentritt et al.^56^ proposed a model where the absorption tails due to electrostatic potential fluctuations γ can be treated like a Gaussian distribution of defects, typically fitted to the low energy tail in PL spectra *I*(*E*), such that
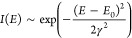
4where *E*_0_ is the average emission energy in the case of fluctuating potentials.^56^ The average potential fluctuation depth γ depends on the total charged defect concentration *N*_t_ and, therefore, can be used as a metric for the absorber film quality, specifically:^53,57^
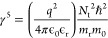
5where *ℏ* is the reduced Planck constant, *m*_0_ is the electron mass and *m*_r_ is the reduced effective mass given by *m*_r_ = ((*m*_e_*m*_h_)/(*m*_e_ + *m*_h_)) with *m*_e_ the effective electron mass and *m*_h_ the effective hole mass. *N*_t_ values of 1.36 × 10^19^ and 2.23 × 10^19^ cm^–3^ were calculated for the large- and small-batch sample films, respectively. Note that *N*_t_ ≠ *N*_D_, as *N*_D_ is a measure of the shallow donor and acceptor defect concentration responsible for radiative recombination within the absorber and may not represent the total defect density *N*_t_, as the presence of deep and/or nonradiative defects is not accounted for. Values for γ for the large batch and small batch were determined to be 40.5 and 49.3 meV, respectively, and are similar to previous results.^33,58,59^ It is apparent that there is less band tailing in the large-batch samples, which results in an average device efficiency of ∼4.2% compared to ∼2.9% for the small-batch devices (Figure S10).

**Table 3:** A List of Optical Parameters Obtained from Luminescence for Large-Batch and Small-Batch CZTSSe Absorber Layers

	large batch	small batch
Δ*E*_PL_ (meV)	29.2	32.5
defect spacing, s (nm)	5.80	5.21
QDAP density, *N*_D_ (cm^–3^)	1.36 × 10^18^	1.87 × 10^18^
electrostatic potential fluctuations γ (meV)	40.5	49.3
total defect density, *N*_t_ (cm^–3^)	1.36 × 10^19^	2.23 × 10^19^

Band tailing due predominantly to the presence of electrostatic potential fluctuations has been proposed to be one prominent mechanism of *V*_OC_ reduction in kesterite solar cells.^60^ Devices made from large-batch nanoparticle inks exhibit a significant increase in *V*_OC_ compared to the small-batch counterparts, with an average value of ∼410 mV compared to ∼340 mV, which can be attributed to larger γ values observed in the small-batch sample films (Figure S10).

Large-batch synthesis methods offer significant advantages compared with small-batch approaches in the realm of photovoltaic device production. Large-batch methods allow for coating a much larger area, accommodating up to 25 substrates of 25 × 75 mm^2^ at once, as opposed to the limited capacity of five 25 × 75 mm^2^ substrates in small-batch processes. The large-batch synthesis, therefore, has a total coating area of 468.75 cm^2^, in contrast to the small-batch synthesis, which coats only 93.75 cm^2^. This scalability translates into improved production efficiency, reducing time and costs while optimizing resource utilization. Large-batch techniques also yielded more uniform and consistent photovoltaic devices, enhancing reliability and predictability. Additionally, the reduced energy and material consumption per unit area coated in large-batch synthesis align with sustainability goals, minimizing the environmental footprint, as qualitatively demonstrated in the LCA section.

## Conclusion

In conclusion, this study investigates scaling-up of kesterite nanoparticle fabrication at the laboratory scale. The study is motivated by an initial life-cycle assessment to achieve a more sustainable design and fabrication of the nanoparticles. Following this, COMSOL simulations were used to optimize fluid flow patterns inside a 500 mL flask, identifying the ideal stirring parameters for CZTS nanoparticle fabrication. Subsequent large-batch synthesis of CZTS nanoparticle inks resulted in smooth, densely packed spherical nanoparticles. These CZTS precursor films were characterized with SEM imagery showing the nanoparticle size to be larger from the small synthesis with a mean size of 22.26 nm compared with that of 15.43 nm of the large-batch-synthesized nanoparticles. We believe the smaller nanoparticle size observed in the large batch to be due to the higher shear rate in the large-batch synthesis, which was discussed in the COMSOL section of the manuscript. The resulting champion devices had PCEs of 5.1% and 3.4% for large and small batches, respectively.

The selenization of the nanoparticle thin films produced high-quality CZTSSe absorbers, as revealed by Raman spectroscopy and X-ray diffraction analysis, showcasing favorable characteristics with close-to-ideal elemental ratios. Notably, the large volume synthesis approach was shown to have lower environmental impacts and a reduced carbon footprint, as demonstrated by the LCA analysis, making it a more environmentally sustainable choice compared to small-batch fabrication methods.

The successful integration of optimized fluid flow patterns, environmentally friendly large-batch synthesis, and high-performance CZTSSe absorbers of thickness ∼1150 nm, with a bandgap of ∼1.1 eV determined with EQE and UV–vis spectroscopy measurements, was made from slot die-coated CZTS nanoparticle precursor films with an optical bandgap of ∼1.5 eV and thickness of ∼900 nm, which demonstrate promising advancements in the field of renewable energy technologies. As we move toward sustainable energy solutions, this study serves as a significant step in the development of cost-effective, scalable, and eco-friendly CZTSSe thin film solar cells, contributing to the realization of a greener and more sustainable future.

Possible future work to follow on and enhance this study would be to investigate the cost and environmental impacts of recycling and disposal of solar cell materials to paint a better picture of the total life-cycle assessment. With respect to the devices, a full economic analysis from a manufacturing perspective would be a good additional study. Finally, a stability analysis for a quantitative efficiency lifetime and performance degradation would be an essential part of our further study.

## Experimental Section

### LCA

An LCA was carried out for the hot injection process used to synthesize the nanoparticles for the nanoparticle ink. To do this, the input materials and electricity consumption values used for the process were noted, and their data were taken from the Ecoinvent database. The amount of waste produced and the composition of this waste was calculated. This information was then organized into the LCA software (SimaPro) which calculated the environmental impact from the inputs according to the method selected, in this case CML-AI v3.08 which investigates 11 impact categories. The results are calculated with respect to a functional unit chosen by the investigator; this was chosen to be 20 mL to allow for comparison between the two different batch sizes. This is achieved by using a flask connected to a multivalve manifold that is used to either purge the reaction chamber with an inert gas or pull a vacuum to evacuate volatiles from the reaction medium (Figure S1). More details are given in the experimental section. Simapro v9.4.0.2 software was used to perform the LCA together with the Ecoinvent v3.8 database. The environmental impact was calculated using the CML-IA baseline v3.08 model and Eu25+3 2000 characterization across 11 impact categories shown in Figure 2. This method has been used in other works^24,25,28^ and is relatively common in PV.^61^

### COMSOL Simulation

COMSOL Multiphysics version 6.0 simulation software was used to study the mixing process of fluid in a 500 mL round-bottom flask. For simplicity, the three-dimensional flask geometry is achieved by 360^◦^ rotation of a two-dimensional *x*–*z* plane around the *z*-axis. Fluid flow equations were solved using stabilized finite element formulations in combination with damped Newtonian methods. Reynolds numbers for rotating magnetic stirrers of length 50 and 65 mm at 200 rpm were calculated to be 8.3 × 10^3^ and 14.0 × 10^3^, respectively.^62^ The Reynolds numbers are significantly greater than unity; therefore, the resulting flow was treated as turbulent. For turbulence modeling, a two-variable *k*–ϵ model was used where modified Navier–Stokes equations calculate the velocity field of the fluid using turbulence viscosity. The fluid in the flask was assumed to be in thermal equilibrium with the vessel wall at 200 ^◦^C. The remainder of the flask was filled with N_2_ at a pressure of 1 atm. The fluid within the flask was assumed as a homogeneous Newtonian fluid obeying the density and viscosity experimentally determined.

### CZTS Large-Batch Nanoparticle Ink Synthesis

For the experimental setup, the reaction flasks were interconnected with a Schlenk line equipped with a vacuum exhaust and nitrogen purge capabilities. Specifically, 1.34 mmol of Cu(acac)_2_, 0.95 mmol of Zn(acac)_2_, 0.75 mmol of Sn(acac)_2_Cl_2_, and 10 mL of OLA were introduced into a 100 mL three-neck flask. In contrast, for the 500 mL flask, the quantities were scaled up to 6.7 mmol of Cu(acac)_2_, 4.75 mmol of Zn(acac)_2_, 3.75 mmol of Sn(acac)_2_Cl_2_, and 50 mL of OLA. Additionally, a 1 M concentration of elemental sulfur (S, 99.98%, Sigma-Aldrich) in OLA was prepared adjacent to the reaction flasks in preparation of the hot injection. These steps ensured consistent molar concentrations across both small- and large-batch synthesis setups with Cu-poor and Zn-rich composition for enhanced solar cell efficiencies. The reaction was carried out at 225 °C for 30 min, and the resulting nanoparticles were precipitated, washed twice with isopropanol (IPA) and toluene, and then dispersed in 1-hexanethiol by sonication, producing CZTS nanoparticle inks with a concentration of approximately 100 mg/mL.

### Kesterite Absorber Fabrication

Thin films of the CZTS precursor were carefully applied to molybdenum-coated SLG substrates using the slot die coating technique. The molybdenum was deposited using a Moorfield minilab 125 sputtering machine with a base pressure of 6 × 10^–8^ mbar then RF sputtering at a rate of 1.2 Å/s for a total thickness of 800 nm. The Mo thin films had a resistivity of 4.20 × 10^–5^ Ω cm and a sheet resistance of 0.5248 Ω/□. To guarantee uniformity and remove residual solvents, the samples were exposed to four cycles of slot die coating and soft baking process at 150 °C for 30 s, followed by a 30 s step at 300 °C in ambient atmosphere. This approach yielded a desired thickness of approximately 900 nm. To convert CZTS nanoparticles into microsized CZTSSe absorbers, precursor layers then underwent tube furnace selenization. To obtain a suitable environment for annealing, the furnace was emptied to 6.0 × 10^–3^ mbar containing 300 mg of Se pellets, and an argon atmosphere of 150 mbar was introduced before increasing the temperature to a constant 500 °C for 20 min as outlined in ref^14^.

### Solar Cell Fabrication

The CZTS absorber films were incorporated into the solar cell devices without interruption, following a specific layering process. To begin, a 50 nm thick CdS buffer layer was applied to the CZTSSe absorber by using chemical bath deposition. Cadmium sulfate and thiourea were used as the cadmium and sulfur sources, respectively, and the pH was adjusted to around 11 with ammonium hydroxide. The transparent oxide layers were then added by magnetron sputtering, which included a 50 nm thick intrinsic ZnO (i-ZnO) layer and a 200 nm thick indium tin oxide (ITO) layer with a sheet resistance of less than 50 Ω/□. For the front contact grid, approximately 100 nm of Au was thermally evaporated through a shadow mask. The total area of the solar cell was around 0.16 cm^2^, which was precisely determined using mechanical scribing with the help of an optical microscope.

### Raman Spectroscopy

Micro-Raman measurements were performed initially at room temperature with a Renishaw inVia confocal Raman microscope (Renishaw plc., Wotton-Under-Edge, UK) in the backscattering configuration using 532 and 488 nm laser excitation with a 50x objective (numerical aperture = 0.50, and beam diameter ≈1 μm). The spectra were acquired at a laser power of 0.15 mW (1.94 × 10^4^ W/cm^2^). Additional micro-Raman spectra were also collected using a HORIBA LabRAM Soleil spectrometer using 0.75 mW laser power at 785 nm and 0.31–0.62 mW power at 405 nm with a 50× objective (0.6 numerical aperture, beam diameter ≈0.6–1.6 μm). Measurements were taken by using multiple averages and at different areas of each sample for consistency.

### Photoluminescence

PL spectra were measured using a HORIBA Jobin Yvon fully automated spectrometer fitted with an InGaAs PMT detector cooled to −30 °C to reduce noise. A 532 nm continuous-wave diode-pumped solid-state (CW-DPSS) laser was used as an excitation source. Low-temperature PL measurements were performed by placing the samples in a Janis SHI-4-2 closed-cycle refrigeration cryostat using compressed He gas coupled to a Lakeshore Model 355 temperature controller.

### XRD Measurement

Table 4 shows the scan parameters for the XRD measurements of both small and large batches and for both CZTS precursor films and CZTSSe absorber layers. For the XRD fitting a pseudo-Voigt curve (eq 6) was used:
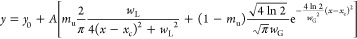
6where *A* > 0: area, *w*_G_, *w*_L_ > 0: Gaussian fwhm, Lorentzian fwhm, *m*_u_: profile shape factor, *y*_0_: *y*-axis offset, and *x*_c_: peak center.

**Table 4:** XRD Scan Parameters

scan parameter	value
measurment range	10–80°
step size	0.0100°
scan speed	3.000°/min
scan resolution	0.0002°
scan type	Bragg–Brentano 2θ/θ
beam current	50 mA
beam voltage	40 kV
incident slit	10 mm
receiving slit	Soller slit 2.5°
X-ray source	Cu Kα (Kβ filter used)
detector	the Rigaku D/teX Ultra 250:1D silicon strip detector
pixel pitch resolution	75 μm

Postfit analysis consists of calculating the integral breadth (β) and fwhm (Γ) for using the Gaussian fwhm (Γ_G_) and Lorentzian fwhm (Γ_L_), the forms of which are given in eqs 7 and 8, respectively. This outlined method is referenced:^41^
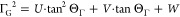
7
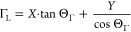
8
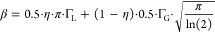
9fwhm (Γ) is computed from Γ_G_ and Γ_L_ as
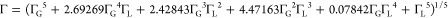
10and the mixing parameter (η) describes the ratio of Lorentzian form to Gaussian form, ranging from 0 to 1 with 1 being fully Lorentzian:
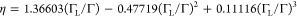
11From the above integral breadth (β) was used in the Scherrer equation (eq 12) to calculate the crystallite size of CZTS nanoparticles in the precursor films as the Wilson equation (eq 13) was used to calculate broadening from microstrain:
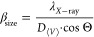
12where *D*_⟨*V*⟩_ refers to the volume-averaged coherently scattering domain size.
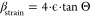
13where ϵ is treated as the volume-averaged microstrain.

### Solar Cell Measurement

To evaluate the current–voltage characteristics of the solar cells, measurements were conducted in a four-point probe configuration employing a Keithley 2400 source meter. Solar cells were subjected to an air mass 1.5 spectrum set at an intensity of 100 mW/cm^2^ from an Abet Technologies Sun 2000 solar simulator. To assess the capacitance–voltage parameters, a potentiostat unit, versastat 4, was employed, operating at a frequency of 100 kHz, and a bias range spanning from 0.5 to −1 V was applied. Furthermore, external quantum efficiency measurements were carried out by using a Bentham PVE300 spectral response system. To ensure accuracy, the system was calibrated using a Si–In GaAs solar cell as a reference point. This comprehensive evaluation process provided valuable information about the performance and characteristics of the solar cells.

## Supporting Information Available

LCA including a glossary for key terms and abbreviations as well as a cost analysis; Raman spectroscopy and UV–vis spectroscopy along with electrical characterization and performance of the large-batch-synthesized solar cells; COMSOL figures demonstrating the sensitivity of the simulation to input parameters (PDF)
