# Supplementary material for: Ecodesign of Kesterite Nanoparticles for Thin Film Photovoltaics at Laboratory Scale
Source: ACS Sustain Chem Eng. 2024 Jul 26;12(31):11613–27. doi: 10.1021/acssuschemeng.4c02841 (PMC11304380; doi:10.1021/acssuschemeng.4c02841)
Supplement: Supplementary file 1 — sc4c02841_si_001.pdf [file sc4c02841_si_001.pdf]

Supporting Information

Michael D. K. Jones,[†] Bethany Willis,[†] Stephen Campbell,[†] Giray Kartopu,[†] Pietro Maiello,[†] Prabeesh Punathil,[†] Wai Ming Cheung,[‡] Elliot Woolley,[¶] Lewis C. R. Jones,[¶] Ochai Oklobia,[§] Adam Holland,[‖] Vincent Barrioz,[†] Guillaume Zoppi,[†] Neil S. Beattie,[†] and Yongtao Qu[*,†]

†*Department of Mathematics, Physics and Electrical Engineering, Northumbria University, Newcastle upon Tyne NE1 8ST, United Kingdom*

‡*Department of Mechanical and Construction Engineering, Northumbria University, Newcastle upon Tyne NE1 8ST, United Kingdom*

¶*Wolfson School of Mechanical, Electrical and Manufacturing Engineering, Loughborough University, Loughborough, Leicestershire, LE11 3TU, UK*

§*Centre for Solar Energy Research (CSER), in the Centre for Integrative Semiconductor Materials (CISM), Faculty of Science and Engineering, Bay Campus, Swansea University, SA1 8EN, UK*

‖*HORIBA UK Limited, Kyoto Close, Moulton Park, Northampton NN3 6FL, UK*

E-mail: y.qu@northumbria.ac.uk

Number of pages: 15

Number of figures: 11

Number of tables: 7

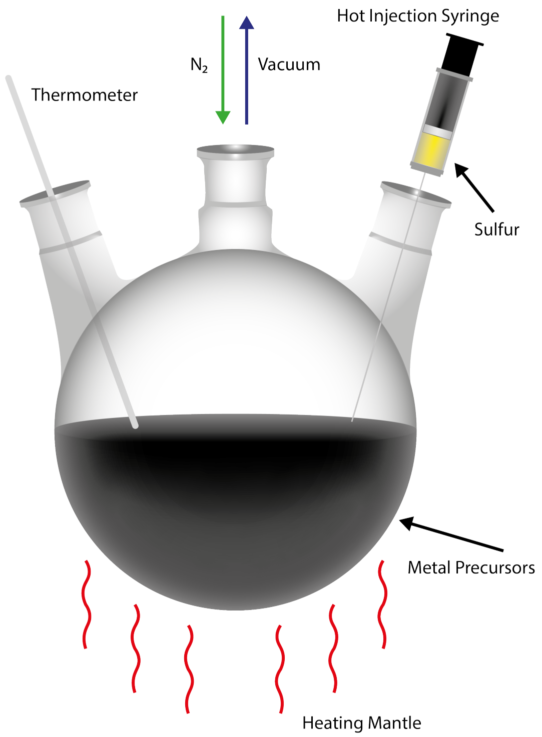

Figure S1: Hot Injection schematic showcasing the experimental setup used in both 100 ml and 500 ml volume synthesis.

Table S1: Showing the input materials for the small batch and large batch for the LCA.

Material	Small Batch	Large Batch
Copper (mg)	355.0	1753.9
Zinc (mg)	252.7	1250.1
Tin (mg)	293.2	1456.1
Sulphur (mg)	320.0	642.0
OLA (Tall, Crude Oil) (mg)	18,940	66,290
Isopropanol (mg)	62496	312480
Toluene (mg)	38925.0	194625.0
Electricity (kWh)	0.322	0.453
Waste Treatment (mg)	108274.5	512000.3

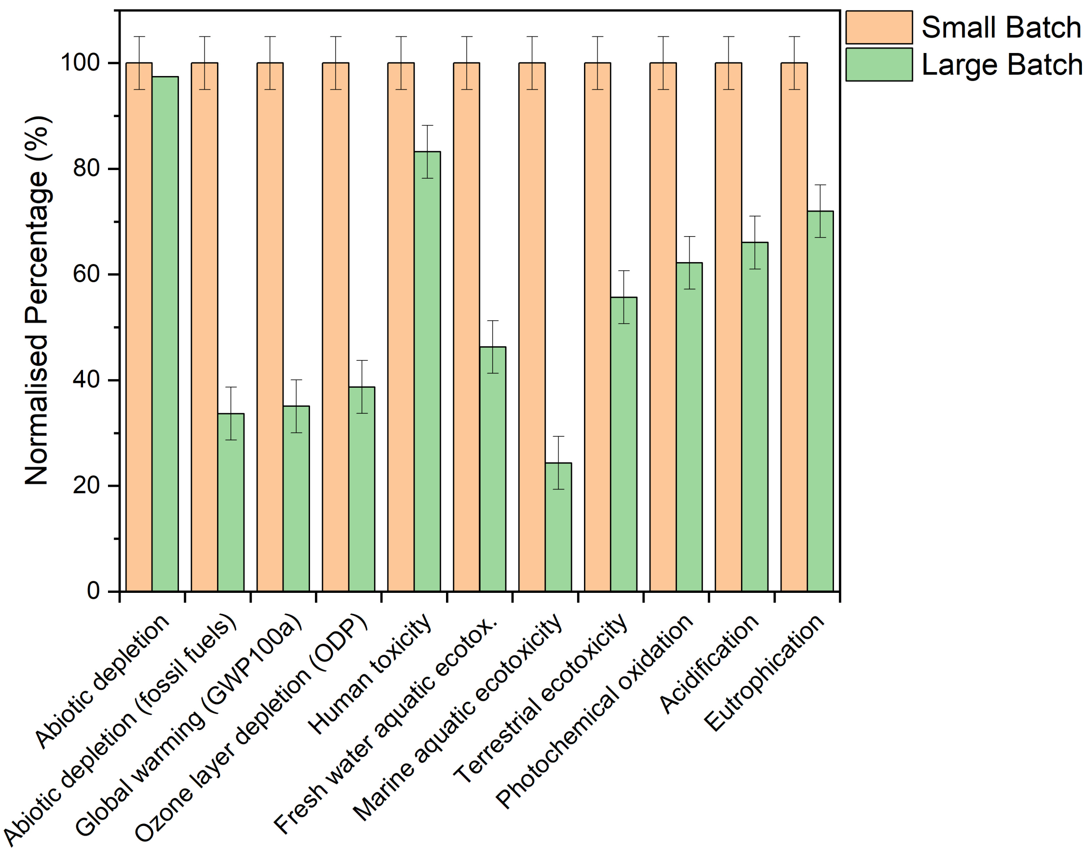

Figure S2: Normalised impact assessment results showing ± 5% variation in input data used in the LCA for small batch and large batch nanoparticle synthesis reactions.

Table S2: LCA raw results for Small Batch vs Large Batch for the nanoparticle synthesis (part 1).

Impact category	Small Batch	Large Batch
Abiotic depletion (kg Sb eq)	9.72E-06	9.47E-06
Abiotic depletion (fossil fuels) (MJ)	1.185275	0.398954
Global warming (GWP100a) (kg CO2 eq)	0.087986	0.030839
Ozone layer depletion (ODP) (kg CFC-11 eq)	5.61E-09	2.17E-09
Human toxicity (kg 1,4-DB eq)	0.347345	0.289043
Fresh water aquatic ecotox. (kg 1,4-DB eq)	0.487621	0.225664
Marine aquatic ecotoxicity (kg 1,4-DB eq)	1900.535	462.2113
Terrestrial ecotoxicity (kg 1,4-DB eq)	0.000366	0.000204
Photochemical oxidation (kg C2H4 eq)	2.06E-05	1.28E-05
Acidification (kg SO2 eq)	0.000461	0.000304
Eutrophication (kg PO4— eq)	0.000219	0.000157

Table S3: LCA raw results for Small Batch vs Large Batch for the nanoparticle ink synthesis (part 1 & 2).

Impact category	Small Batch	Large Batch
Abiotic depletion (kg Sb eq)	1.04E-05	1.01E-05
Abiotic depletion (fossil fuels) (MJ)	7.580585	6.473596
Global warming (GWP100a) (kg CO2 eq)	0.368788	0.289847
Ozone layer depletion (ODP) (kg CFC-11 eq)	1.35E-08	8.74E-09
Human toxicity (kg 1,4-DB eq)	1.056734	0.992251
Fresh water aquatic ecotox. (kg 1,4-DB eq)	4.719408	4.451085
Marine aquatic ecotoxicity (kg 1,4-DB eq)	26168.18	24716.33
Terrestrial ecotoxicity (kg 1,4-DB eq)	0.00063	0.000406
Photochemical oxidation (kg C2H4 eq)	0.000164	0.000153
Acidification (kg SO2 eq)	0.001219	0.001008
Eutrophication (kg PO4— eq)	0.0004	0.000321

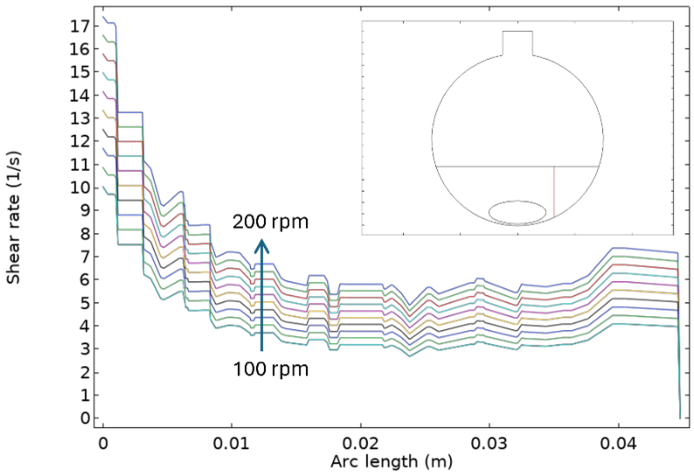

Figure S3: Shear rate along a line indicated in red in the fluid of the large 500 ml flask (inset) over a range of magnetic stirrer speeds from 100 to 200 rpm.

Table S4: LCA raw results for different electricity mixes for the nanoparticle ink synthesis.

Impact category	UK, Small Batch	Renewable Small Batch	UK, Large Batch	UK, Large Batch Nanoparticles into Small Batch Ink	Large Batch Nanoparticles into Small Batch Ink
Abiotic depletion (kg Sb eq)	1.04E-05	1.04E-05	1.01E-05	1.01E-05	1.02E-05
Abiotic depletion (fossil fuels) (MJ)	7.580585	6.1406	6.473596	6.077377	6.785205
Global warming (GWP100a) (kg CO2 eq)	0.368788	0.273085	0.289847	0.263514	0.311024
Ozone layer depletion (ODP) (kg CFC-11 eq)	1.35E-08	7.66E-09	8.74E-09	7.14E-09	1E-08
Human toxicity (kg 1,4-DB eq)	1.056734	1.038695	0.992251	0.987288	0.998257
Fresh water aquatic ecotox. (kg 1,4-DB eq)	4.719408	4.697026	4.451085	4.444926	4.457272
Marine aquatic ecotoxicity (kg 1,4-DB eq)	26168.18	26114.19	24716.33	24701.48	24729.47
Terrestrial ecotoxicity (kg 1,4-DB eq)	0.00063	0.000575	0.000406	0.000391	0.000466
Photochemical oxidation (kg C2H4 eq)	0.000164	0.000152	0.000153	0.00015	0.000156
Acidification (kg SO2 eq)	0.001219	0.000987	0.001008	0.000944	0.001061
Eutrophication (kg PO4— eq)	0.0004	0.000325	0.000321	0.0003	0.000338

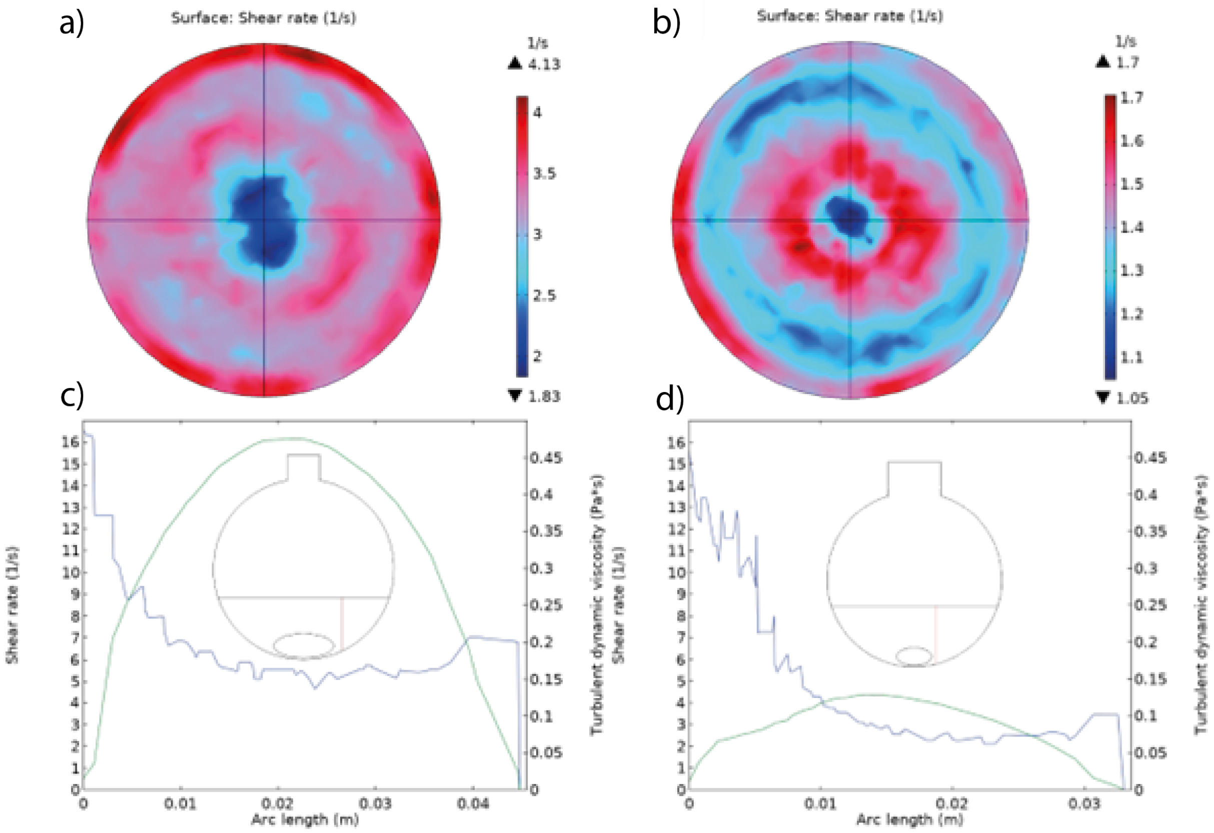

Figure S4: 2-D simulation of the fluid surface in (a) 500 ml flask with 50 mm stirrer and (b) 100 ml flask with 20 mm stirrer. The shear rate is reasonably uniform over the fluid surface in the 500 ml flask and approximately double the magnitude calculated for the 100 ml flask. Corresponding shear rate and turbulent dynamic viscosity are shown as a function of arc length for the (c) 500 ml and (d) 100 ml batches.

Table S5: XRD psuedo-Voigt fit parameters determined for the 3 main CZTS peaks for small batch and large batch. The fitted parameters are: A - area, Γ_L - Lorentzian FWHM, Γ_G - Gaussian FWHM, Γ - FWHM, η - mixing parameter (1.0 being a fully Lorentzian profile, 0.0 fully Gaussian profile), β - Integral Breadth, Domain size - Determined with the Scherrer equation, Strain - determined using the Wilson equation.

A	Γ_L	Γ_G	2θ	Γ	η	β	Domain Size	Strain
8810.15	0.70	0.10	28.51	0.71	0.98	1.08	14.75	1.06
3135.96	0.72	0.10	47.47	0.74	0.99	1.12	15.05	0.64
1868.35	0.85	0.10	56.25	0.87	0.99	1.33	13.14	0.62
2673.51	0.63	0.10	28.49	0.64	0.98	0.97	16.41	0.95
872.62	0.60	0.10	47.45	0.62	0.98	0.93	18.09	0.53
482.66	0.60	0.10	56.25	0.62	0.98	0.93	18.78	0.44

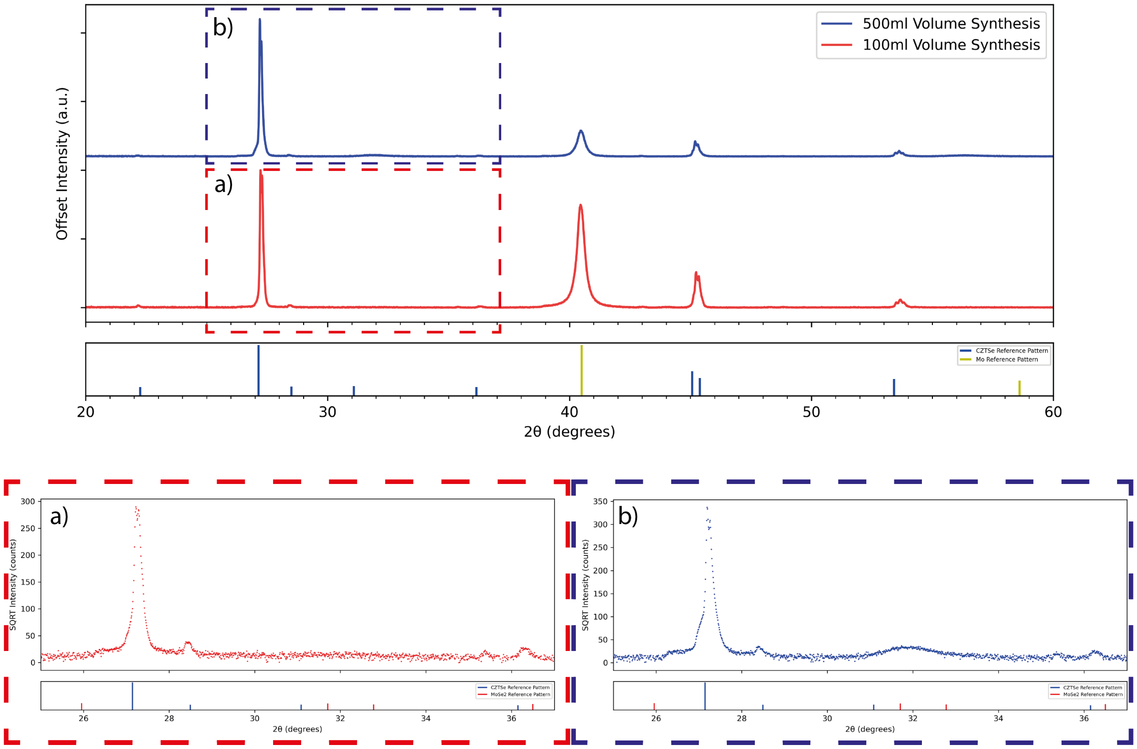

Figure S5: X-ray diffractogram showing the relative peak intensities of the selenised CZTSSe crystalline phases found in small and large batch synthesised. XRD diffractograms show the main peak phases at miller indices (112), (220) and (312) against the reference pattern for pure CZTSe (PDF 052-0868) with comparable intensities between 100 ml and 500 ml synthesised inks. Square root subplots of a) 100 ml synthesis and b) 500 ml synthesis between 2θ angles 25° and 37° highlight smaller key peaks, which match with either CZTSSe or $MoSe_2$ diffraction patterns. It is worth noting that both samples have a low intensity peak at 35.5° corresponding to the Wurtzite phase of CZTSSe (Zhang et al., 2018, Scientific Reports).

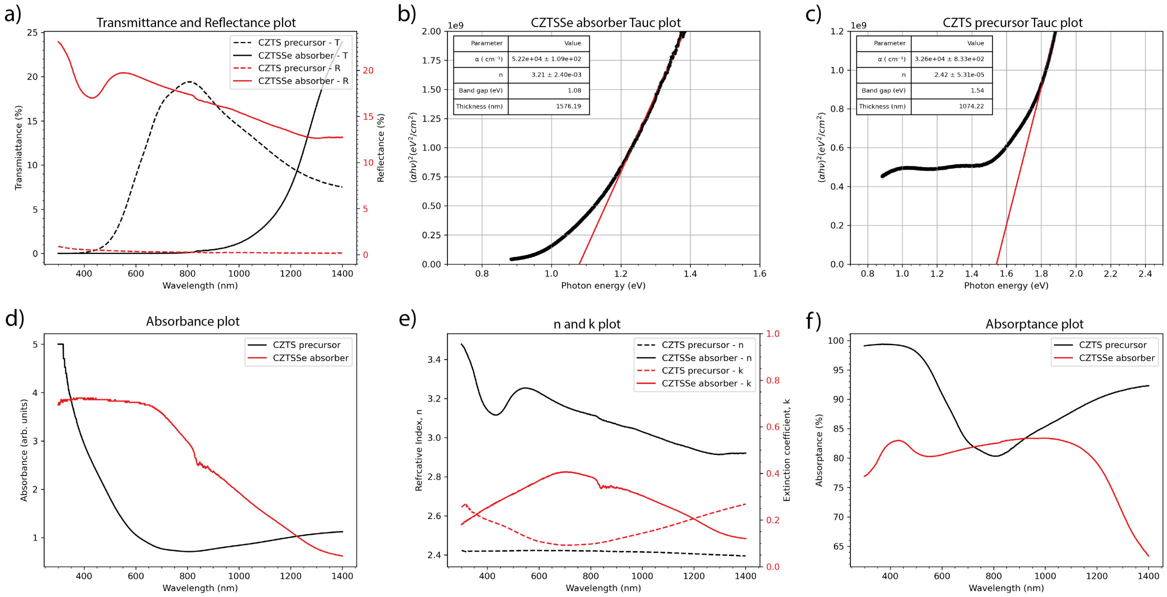

uv

Figure S6: UV-Visible spectroscopy of large bacth synthesised nanoparticle CZTS precursor films and CZTSSe absorber thin films. Subplots a) Transmittance and Reflectance plot, b) CZTSSe absorber Tauc plot, c) CZTS precursor Tauc plot, d) Absorbance plot, e) n and k refractive index plot and f) Absorptance plot.

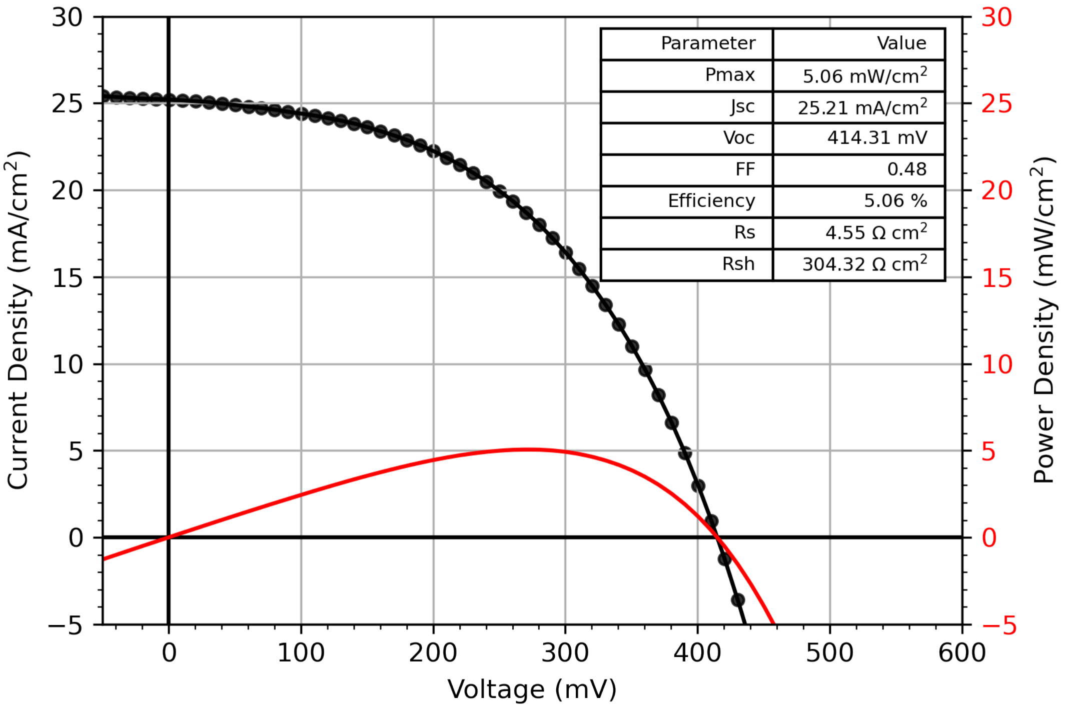

Figure S7: Current Voltage characteristic of a champion cell from the large synthesis

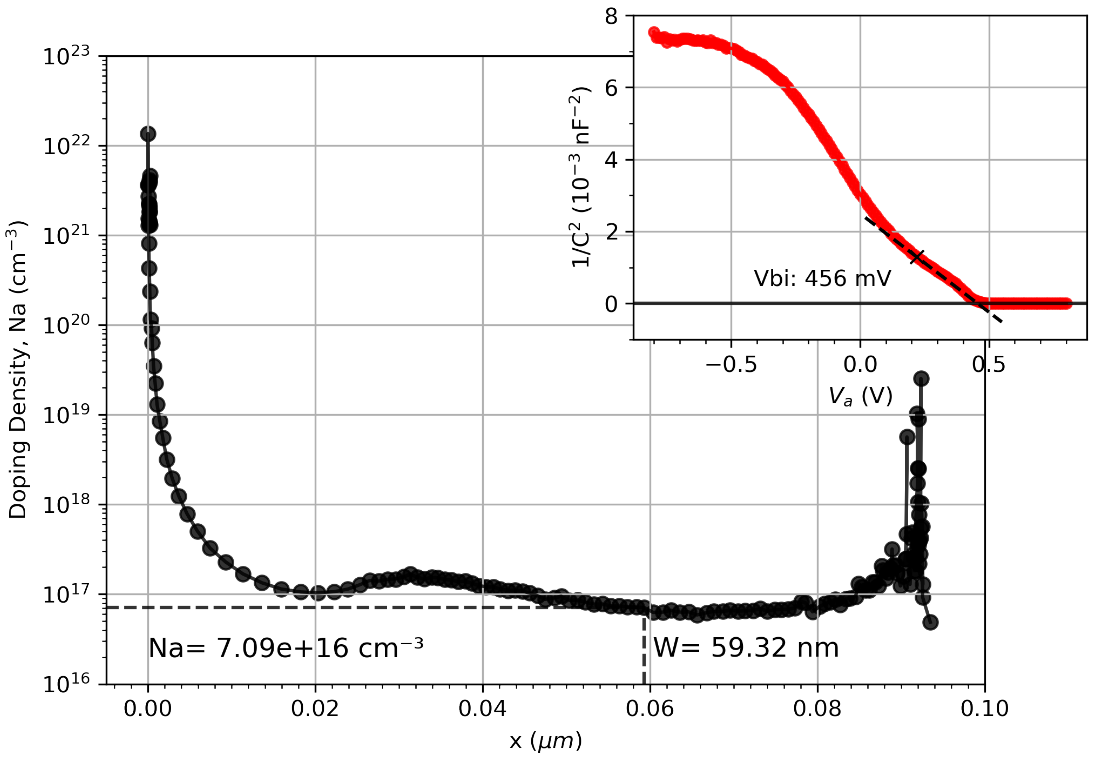

Figure S8: Capacitance-Voltage characteristic of a large batch synthesised champion cell

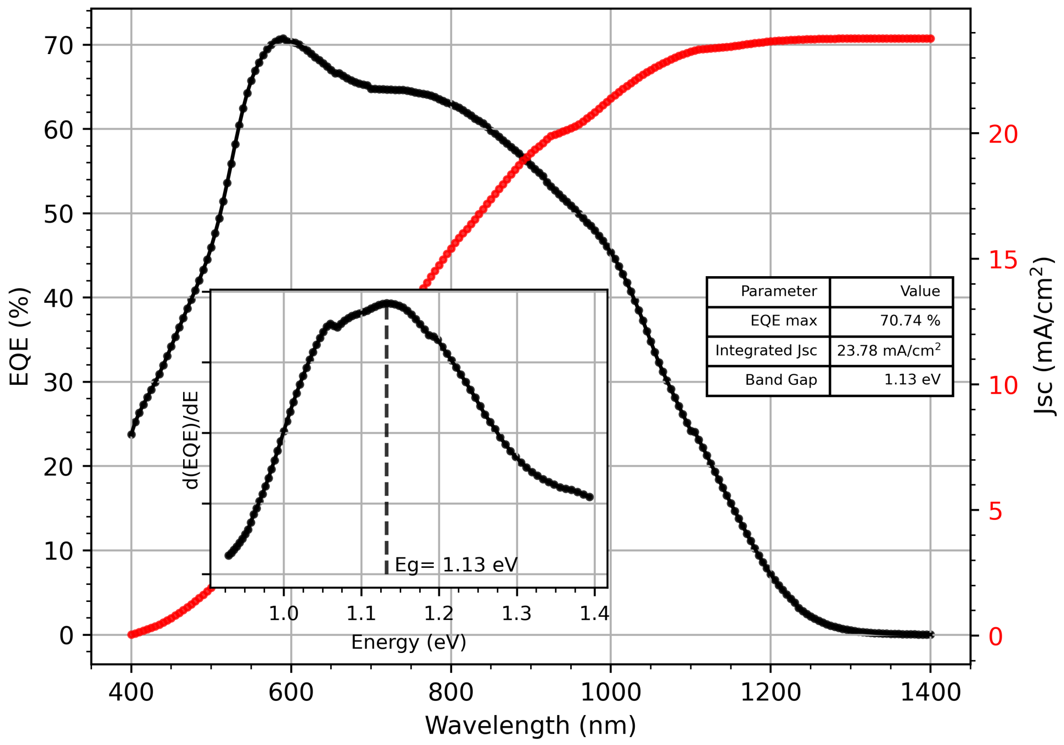

Figure S9: External quantum efficiency measurement and integrated short-circuit current of a large batch synthesised champion cell

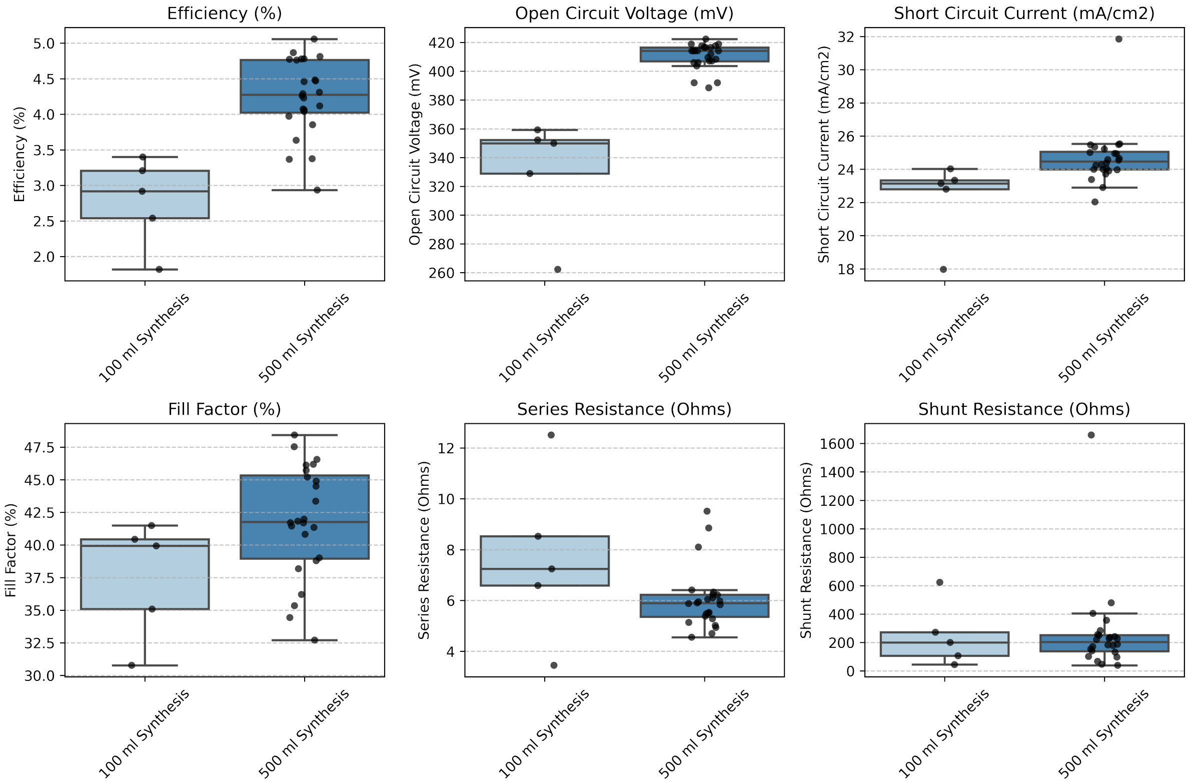

Figure S10: A comparison between small batch and large batch of electronic device parameters obtained through the current-voltage measurements

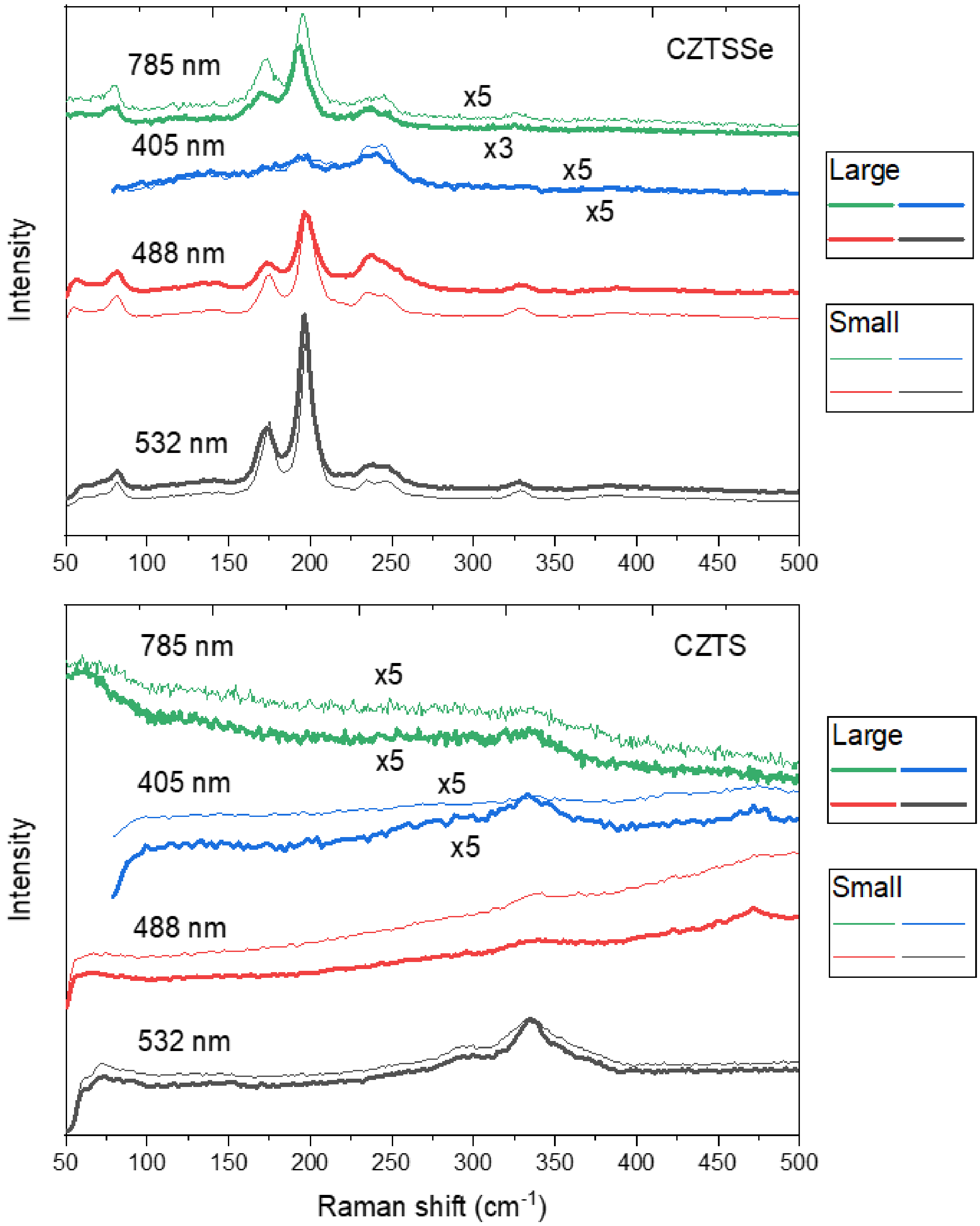

Figure S11:Raman spectra of (a) CZTS and (b) CZTSSe films excited at various wavelengths. Secondary phases observable in CZTS spectra, namely bands 297 cm^{-1} with 532 nm excitation due to Cu_3SnS_4 (CTS) and 473 cm^{-1} with 488 and 405 nm excitations due to $Cu_{2-x}S$, are eliminated after selenization, viz. CZTSSe films.

Table S6: LCA Glossary

Cradle-to-Gate	Considers the life cycle stages associated with the mining of the raw materials, transport to the manufacturing location and the manufacturing of the product – in this case the nanoparticle ink.
Goal and Scope	The introduction to the LCA which provides answers to the who, what, where, when, why and how of the LCA study.
Global market	These are types of inputs taken from the Ecoinvent database which take the average of input and output emissions from all over the world for each input and include the transportation of these inputs.
CML-IA	A type of LCA model which investigates a certain set of environmental impact categories, different models investigate different impact categories. Each of the impact categories associated with this method are explained in more detail below.
Eu25+3 2000	The characterisation method is used to group the impact of different materials and emissions relative to a reference substance. For example, Global warming potential (GWP) includes CO2, CO, CH4, N2O, etc which are all expressed in terms of kg CO2 equivalent.
Abiotic depletion	Measured in kg Sb eq. (kilograms of antimony equivalent), describes how the quantity of raw resources (not fossil fuels) is depleted as a result of the product/process being investigated. More scarce raw materials will have a greater contribution to this impact category than more abundant materials.
Abiotic depletion (fossil fuels)	Measured in MJ (megajoules), describes how the quantity of fossil fuel resources extracted from the Earth are depleted.
Global warming potential	Measured in kg CO2 eq. (kilograms of carbon dioxide equivalent), describes the quantity of greenhouse gasses released into the atmosphere with a time horizon of 100 years (which is why it is expressed as GWP100a in data tables and figures).
Ozone layer depletion	Measured in kg CFC-11 eq. (kilograms of trichlorofluoromethane equivalent), relates to the emissions that are released which cause damage and degradation to the stratospheric ozone layer resulting in increased UV-B radiation reaching the Earth's surface. This radiation is known to cause harm to plants, humans and animals. CFCs are one of the most familiar chemicals when discussing ozone layer depletion.

Human toxicity	Measured in kg 1,4-DB eq. (kilograms of 1,4-dichlorobenzene equivalent), describe the fate, exposure and effects of toxic matter on humans from emissions and substances.
Freshwater aquatic ecotoxicity	Measured in kg 1,4-DB eq. (kilograms of 1,4-dichlorobenzene equivalent), describes the fate, exposure and effects of toxic matter on freshwater ecosystems due to emissions and contamination of substances to the air, soil and water.
Marine aquatic ecotoxicity	Measured in kg 1,4-DB eq. (kilograms of 1,4-dichlorobenzene equivalent), describes the fate, exposure and effects of toxic matter on marine ecosystems due to emissions and contamination of substances to the air, soil and water.
Terrestrial ecotoxicity	Measured in kg 1,4-DB eq. (kilograms of 1,4-dichlorobenzene equivalent), describes the fate, exposure and effects of toxic matter on terrestrial ecosystems due to emissions and contamination of substances to the air, soil and water.
Photochemical oxidation	Measured in C2H4 eq. (kilograms of ethylene equivalents), relates to the quantity of reactive substances forming in the atmosphere as a result of released emissions. An example of this is "summer smog". This can affect humans, plants and animals.
Acidification	Measured in kg SO2 eq. (kilograms of sulphur dioxide equivalent), is related to the fate and increased deposition of acidic matter in air, water and land due to emissions. This can negatively affect materials, land and aquatic ecosystems.
Eutrophication (sometimes known as nutrification)	Measured in kg PO4— eq. (kilogram of phosphate equivalent), describes the quantity of excess nutrients in the environment due to emissions contaminating air, water and soil. These excess nutrients cause excessive growth of plants and algae resulting in oxygen depletion in the ecosystem and death of organisms making the ecosystem inhabitable.

Table S7: Cost analysis of small batch vs large batch synthesis.

**Cost Analysis**	**Cost of small batch (£, 20 mL ink)**	**Cost of large batch (£, 100 mL ink)**
Copper (mg) 50 g @ £165 (Sigma-Alrich)	1.16 (355.0 mg)	5.80 (1775.0 mg)
Tin (mg) 5 g @ £105 (Sigma-Alrich)	6.09 (293.2 mg)	30.45 (1466.0 mg)
Zinc (mg) 25 g @ £278 (Sigma-Alrich)	2.89 (252.7 mg)	14.45 (1263.5 mg)
Sulphur (mg) 50 g @ £94.6 (Sigma-Alrich)	0.61 (0.32 g)	1.22 (0.64 g)
OLA (mg) 1.5 kg @ £229 (Sigma-Alrich)	2.89 (20 mL)	10.11 (70 mL)
Isopropanol (mg) 2.5 L @ £135 (Fisher scientific)	4.32 (80 mL)	21.60 (400 mL)
Toluene (mg) 1 L @ £42 (Fisher scientific)	1.89 (45 mL)	9.45 (225 mL)
**Total raw materials**	**19.85**	**93.08**
**Equipment, include glassware, heat mantle, syringe, etc.** Increase fabrication times will spread the equipment cost and large batch will spread the cost even thinner.	**1073.33**	**1311.50**
**Electricity (kWh)** 26 p/kWh (Business Electricity Rates for large business)	**0.08 (0.322 kWh)**	**0.12 (0.453 kWh)**
**Labour** @£20.45 per hour	**163.60**	**163.60**
**Waste disposal** The disposal of 1 lite of CZTS waste would be £3.00. 105 mL waste and 525 mL waste are generated in for small scale and large scale respectively. Hazardous waste is collected monthly. The costs of the monthly collection are as follows: Transport £100; Administration £65; Technical Support £55	**220.32**	**221.58**
**Total cost**	**1477.28**	**1789.88**
**Normalised Cost** (per 20 mL ink)	**1477.28**	**357.98**
